# GRADE-ADOLOPMENT of clinical practice guidelines and creation of clinical pathways for the primary care management of chronic respiratory conditions in Pakistan

**DOI:** 10.1186/s12890-023-02409-4

**Published:** 2023-04-17

**Authors:** Russell Seth Martins, Hawra Hussain, Maryam Chaudry, Nashia Ali Rizvi, Mohsin Ali Mustafa, Bushra Ayub, Salima Saleem Aamdani, Alina Abdul Rehman, Alina Pervez, Sarah Nadeem, Rameesha Khalid, Akbar Shoukat Ali, Shayan Shahid, Ali Bin Sarwar Zubairi, Adil H. Haider, Muhammad Irfan

**Affiliations:** 1 Center for Clinical Best Practices, Clinical and Translational Research Incubator (CITRIC), Aga Khan University, Karachi, 74800 Pakistan; 2 Medical College, Aga Khan University, Karachi, 74800 Pakistan; 3 Learning Research Centre, Patel Hospital, Karachi, 75300 Pakistan; 4 Department of Medicine, Aga Khan University, Karachi, 74800 Pakistan; 5 Section of Endocrinology, Department of Medicine, Aga Khan University, Karachi, 74800 Pakistan; 6 Section of Pulmonology, Department of Medicine, Aga Khan University, Karachi, 74800 Pakistan

**Keywords:** Asthma, Chronic obstructive pulmonary disease, Idiopathic pulmonary fibrosis, Bronchiectasis, Pakistan, Lower-middle-income country

## Abstract

**Introduction:**

In Pakistan, chronic respiratory conditions contribute a large burden of morbidity and mortality. A major reason for this is the lack of availability of local evidence-based clinical practice guidelines (EBCPGs) in Pakistan, particularly at the primary care level. Thus, we developed EBCPGs and created clinical diagnosis and referral pathways for the primary care management of chronic respiratory conditions in Pakistan.

**Methods:**

The source guidelines were selected by two local expert pulmonologists after a thorough literature review on PubMed and Google Scholar from 2010 to December 2021. The source guidelines covered idiopathic pulmonary fibrosis, asthma, chronic obstructive pulmonary disorders, and bronchiectasis. The GRADE-ADOLOPMENT process consists of three key elements: adoption (using recommendations as is or with minor changes), adaptation (effective context-specific changes to recommendations) or additions (including new recommendations to fill a gap in the EBCPG). We employed the GRADE-ADOLOPMENT process to adopt, adapt, adopt with minor changes, or exclude recommendations from a source guideline. Additional recommendations were added to the clinical pathways based on a best-evidence review process.

**Results:**

46 recommendations were excluded mainly due to the unavailability of recommended management in Pakistan and scope beyond the practice of general physicians. Clinical diagnosis and referral pathways were designed for the four chronic respiratory conditions, explicitly delineating the role of primary care practitioners in the diagnosis, basic management, and timely referral of patients. Across the four conditions, 18 recommendations were added (seven for IPF, three for bronchiectasis, four for COPD, and four for asthma).

**Conclusion:**

The widespread use of the newly created EBCPGs and clinical pathways in the primary healthcare system of Pakistan can help alleviate the morbidity and mortality related to chronic respiratory conditions disease in the country.

**Supplementary Information:**

The online version contains supplementary material available at 10.1186/s12890-023-02409-4.

## Introduction

Chronic respiratory diseases are a leading cause of morbidity, mortality, and disability worldwide [1]. Chronic obstructive pulmonary disease (COPD) is the 3rd leading cause of mortality worldwide [2]. In the United States (US), the estimated prevalence of asthma and COPD in adults is 9.2% and 6.5%, respectively [3]. In Pakistan, a South Asian lower-middle-income country (LMIC), the prevalence of asthma in adults may be as high as 11.3% [4] and COPD as high as 13.8% [5]. Notable risk factors for chronic respiratory illnesses in Pakistan include exposure to environmental pollution, carbon emissions, excessive indoor air conditioner use, mosquito coil use, and high rates of smoking [6].

Evidence-based clinical practice guidelines (EBCPGs) assist the diagnosis and management of chronic respiratory conditions. Currently, most EBCPGs followed globally, such as the Global Initiative for Chronic Obstructive Lung Disease (GOLD) guidelines [7] and the Global Initiative for Asthma (GINA) guidelines [8], are created by institutions in high-income countries in the West. LMICs, like Pakistan, usually lack the research infrastructure and financial resources to independently created EBCPGs de novo for their own healthcare context [9], and instead adapt those already developed by Western countries. However, the application of such EBCPGs in Pakistan presents a problem, as the landscape of respiratory conditions differs in many aspects. These include but are not limited to epidemiology [4], genetic variability [10], healthcare financing and access [11], triggers and risk factors [12], socio-cultural influences [13], disease related awareness [14]. In addition, there is a lack of specialist pulmonologists in Pakistan, particularly in rural areas. Thus, the role of the general practitioner (GP) in the diagnosis and management of chronic respiratory diseases [15] is especially important in Pakistan.

The GRADE-ADOLOPMENT process [9], developed by GRADE (Grading of Recommendations Assessment, Development, and Evaluation), is a globally accepted and implemented process of EBCPG “adolopment”. “Adolopment” describes a combination of adoption (verbatim use), adaptation (contextual modifications), and de novo development/addition, thus leveraging the benefits of pre-existing high-quality EBCPGs while ensuring local appropriateness. GRADE provides a systematic process to assess the certainty of evidence and to make recommendations based on two standardized tables. The Summary of Findings (SoF) table, which presents a summary of findings and certainty of evidence rating for each included outcome, feeds into the evidence-to-decision (EtD) table. The EtD table serves as a template to summarize the quality of available evidence, the judgements that influence the quality rating, and the effects of different management action plans on the outcomes of choice. This helps guide panelists to make decisions regarding the need for contextual modifications of individual recommendations within an EBCPG [16]. GRADE-ADOLOPMENT has been used in countries and regions across the world, including Saudi Arabia [9], Australia [17], Tunisia [18], the Eastern Mediterranean region [19], the Asia-Pacific region [20], Mexico [21], Pakistan [22], and the United Kingdom [23].

While the Pakistan Chest Society (PCS), established 1983, is involved in the creation of local EBCPGs for management of common respiratory diseases [24], the processes involved in the development of these EBCGPs are not explicitly delineated. Consequently, there is immense need for local respiratory EBCPGs to be developed following a transparent, standardized process that makes use of existing available EBCPGs with appropriate context-specific modifications. Such EBCPGs would bring the healthcare system of Pakistan a step closer to achieving optimal health outcomes in respiratory diseases and would have high credibility, by virtue of their transparent development processes. Thus, we aimed to employ the GRADE-ADOLOPMENT process to develop local EBCPGs and create clinical diagnosis and referral pathways for the primary care management of chronic respiratory conditions in adults by GPs in Pakistan.

## Methodology

### Setting

This process was conducted at the CITRIC (Clinical and Translational Research Incubator) Center for Clinical Best Practices (CCBP) at the Aga Khan University (AKU), Pakistan. The AKU is a private sector, not-for‐profit hospital in Pakistan, and is also the country’s leading healthcare and biomedical research facility [25].

The CITRIC CCBP at AKU is tasked with the adaptation and development of EBCPG and care pathways to standardize and improve healthcare in Pakistan for the adult population. The GRADE-ADOLOPMENT processes described in this study have been implemented by the CCBP, in collaboration with the expertise of the Section of Pulmonology (within the Department of Medicine) at AKU and the GRADE-USA working group, in the development of EBCPGs for the management of chronic respiratory conditions by GPs in Pakistan. The decision to create EBCPGs for GPs rather than specialist pulmonologists is due to the lack of specialists in Pakistan [26].

### Study team

The study team is comprised of the CCBP research staff (who are proficient in GRADE methodology and in the development of EBCPGs) as well as pulmonology faculty led by Section Head of Pulmonology at AKU.

### Source guideline selection

The CCBP team first requested the Section of Pulmonology to identify the most common and most impactful respiratory conditions based on their clinical expertise and experience. After finalizing these conditions, the selection process for appropriate and suitable source guidelines was initiated. The source guideline is the single, original, “parent” EBCPG that undergoes the GRADE-ADOLOPMENT process in the development of a local EBCPG. Two local expert pulmonologists appraised various guidelines after an extensive literature review on PUBMED and Google Scholar from 2010 to December 2021, considering criteria such as scope, local familiarity and applicability, rigor, and credibility of creating bodies for each EBCPG. The following were selected as source EBCPGs:

1. *British Thoracic Society Guideline for Bronchiectasis in Adults* [27].
2. *An Official American Thoracic Society/European Respiratory Society/Japanese Respiratory Society/Latin American Thoracic Association Clinical Practice Guideline: Treatment of Idiopathic Pulmonary Fibrosis. An Update of the 2011 Clinical Practice Guideline* [28].
3. *Global Initiative for Asthma Strategy 2022: Executive Summary and Rationale for Key Changes* [29].
4. *Global Initiative for Chronic Obstructive Lung Disease (2022 Edition)* [30].

### Guideline review

**Fig. 1:**
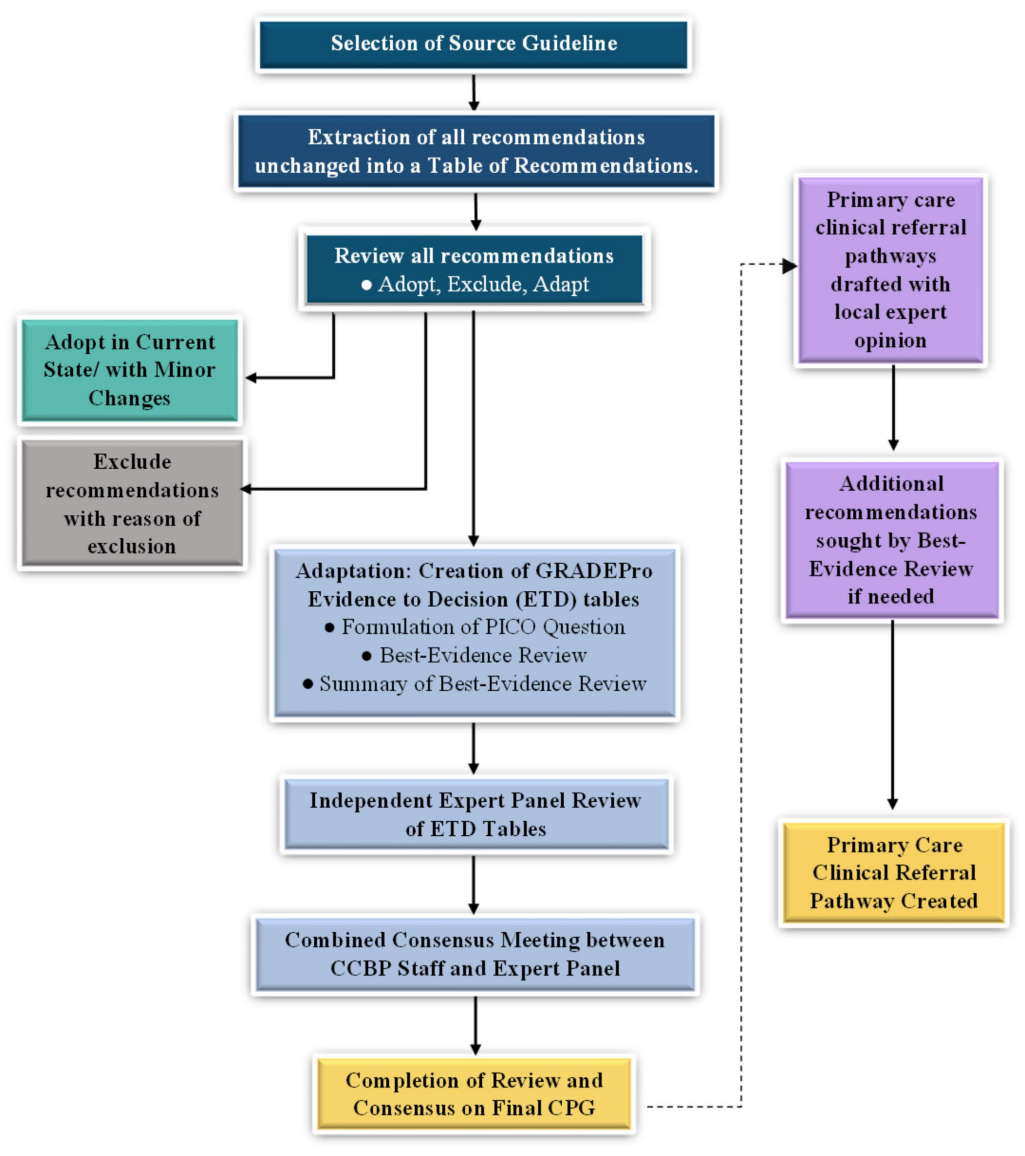
Process of GRADE-ADOLOPMENT and Primary Care Clinical Pathway Creation

Figure 1 delineates the “adolopment” process used in our study. First, a Table of Recommendations (ToR) was created by extracting and compiling all recommendations mentioned in the source EBCPG. Two senior attending pulmonologists reviewed the ToR independently and marked each recommendation as either “*Adopt*,” “*Adapt”* or “*Exclude*.” Discrepancies were settled in consensus with the Section Head of Pulmonology. Recommendations marked “*Adopt*” were incorporated as is or with minor changes into the recommendations of our local EBCPG, while recommendations marked “*Exclude*” were omitted from the final local EBCPG. Exclusion was based on the recommendation pertaining to pediatric or inpatient management, or if the recommendation was deemed irrelevant to the local Pakistani context. Other reasons for exclusion were required to be explained by the reviewers. Recommendations marked “*Adapt*” were deemed to warrant additional review and revision via the GRADE-ADOLOPMENT process (detailed in the **Supplement**) before incorporation into the local EBCPGs.

Our “adolopment” process (Fig. 1) had an important difference from the one described originally [31]: recommendations that were deemed to require only minor and straightforward changes prior to adoption and which did not alter the meaning of recommendation but only provided supporting information/clarification, were not subjected to the complete adaptation process consisting of EtD tables and expert panel review. In the original GRADE-ADOLOPMENT process, adaptation is performed if content changes are deemed potentially necessary for a specific recommendation. This is conducted via the creation of an EtD table and subsequent panel review. In our study however, after a thorough review of the recommendations, it was deemed by the experts that no recommendation needed adaptation. Lastly, if any gaps were found during the formation of the clinical diagnosis and referral pathways, additional recommendations were sought through the best-evidence systematic review process. This ideally included use of recommendations from pre-existing EBCPGs other than the source EBCPG. The evidence used to develop this specific recommendation was reviewed by the experts. If no pre-existing suitable pre-existing EBCPGs were found, recommendations were created and added to the clinical diagnosis and referral pathway using peer-reviewed evidence from reputable information sources, such as Medscape. If the best-evidence systematic review process yielded absolutely no citable evidence, additions were made based on expert consensus.

Two focus group discussions (FGDs) were conducted to identify challenges faced throughout the entire GRADE-ADOLOPMENT process and to explore corresponding solutions. These FGDs were moderated by one of the authors who was selected due to their holistic experience and qualifications. These qualifications included a medical degree (Bachelor of Medicine, Bachelor of Surgery), a postgraduate public health degree (Master’s in Public Health), and certified training in GRADE methodology. In addition, the moderator also had extensive prior experience and training in conducting FGDs during their postgraduate degree. The FGDs were conducted in English, as all participants possessed complete professional competency in the English language. None of the participants disclosed any conflicts of interest with regards to participating in the FGDs.

The two FGDs each included six participants, including the CCBP staff and the Section Head of Pulmonology, a moderator, and a scribe. The scribe did not participate in the discussion but took notes and recorded the audio of the discourse. Each FGD was one hour-long FGDs. Experts were given the opportunity to first brainstorm challenges and solutions independently based on the challenges faced during the GRADE-ADOLOPMENT process for local EBCPG creation. These challenges and solutions were then discussed within the FGD. At the end of each session, a feedback form was given to elicit additional anonymous feedback. After both FGDs were concluded, the audio recordings were transcribed and annotated with the scribes’ notes. Two members of the research team went through the transcriptions and identified challenges and solutions. Each challenge was decided as per consensus opinion to be either a major or minor challenge. The CCBP team then categorized the final list of specific challenges within broad themes, and their corresponding solutions were presented alongside them.

### Timeline of creation of chronic pulmonary disease EBCPGs for Pakistan

The methodology described in this study was carried out according to the following timeline, spanning October 2021-October 2022:

- Source Guideline Selection: October 2021-March 2022 (about 3 weeks for each of the four guidelines).
- Creation of Table of Recommendations: March 2022 (3 weeks for all four guidelines).
- Review of Table of Recommendations: April - May 2022 (for all four guidelines).
- Creation of final Pakistani Chronic Pulmonary Disease EBCPG: June-July 2022 (all four guidelines).
- Focal group discussion: July 2022 (2 weeks to determine the challenges and solution).
- Primary care clinical diagnosis and referral pathways draft creation: August 2022 (2 weeks for all four guidelines).
- Additional recommendations based on best evidence review: September 2022 (2 weeks for all additional recommendations).
- Primary care clinical diagnosis and referral pathways finalized: October 2022 (2 weeks for all four pathways).

## Results

The total number of recommendations within the source EBCPGs were 24 for idiopathic pulmonary fibrosis (IPF), 59 for bronchiectasis, 93 for COPD and 102 for asthma. Minor changes to recommendations were made for the IPF and bronchiectasis EBCPGs (Tables 1 and 2). Across the four conditions, a total of 46/278 (IPF: 2/24; bronchiectasis: 1/59; COPD: 12/93; and asthma: 31/102) recommendations were excluded, and the recommendations with the reason for exclusion can be found in **Supplement Sects. 3–6**. The complete EBCPG can be found in the **Supplementary Materials**.

Primary care clinical diagnosis and referral pathways for the four chronic respiratory conditions are depicted in **Supplementary Figs.** 1–4. The flowcharts focused on appropriate assessment, counseling, preliminary management, and timely referral. Additional recommendations were included for the IPF (Table 1), bronchiectasis (Table 2), COPD (Table 3), and asthma (Table 4) clinical pathways to cover any clinical gap found.

**Table 1:** Recommendations adopted with minor changes and those added to the clinical referral pathway for Primary Care Management of Idiopathic Pulmonary Fibrosis AAFP: American Academy of Family Physicians; ATS/ERS: American Thoracic Society/ European Respiratory Society; BAL: Bronchoalveolar Lavage; GERD: Gastroesophageal Reflux Disease; HRCT: High Resolution Computed Tomography; ILD: Interstitial Lung Disease; IPF: Idiopathic Pulmonary Fibrosis; TBBX: Transbronchial Biopsy UIP: Usual Interstitial Pneumonia

Recommendations adopted with Minor Changes		
Original Recommendations	Recommendations Adopted with Minor Changes	
“For patients with newly detected ILD of apparently unknown cause who are clinically suspected of having IPF and have an HRCT pattern of probable UIP, indeterminate for UIP, or an alternative diagnosis, we suggest cellular analysis of their BAL fluid (conditional recommendation, very low quality of evidence).” [28]	For patients with newly detected ILD of apparently unknown cause who are clinically suspected of having IPF and do not have an HRCT pattern of definite UIP, we suggest cellular analysis of their BAL fluid (conditional recommendation, very low quality of evidence).	
“For patients with newly detected ILD of apparently unknown cause who are clinically suspected of having IPF and have an HRCT pattern of probable UIP, indeterminate for UIP, or an alternative diagnosis, we suggest SLB (conditional recommendation, very low quality of evidence).” [28]	For patients with newly detected ILD of apparently unknown cause who are clinically suspected of having IPF and do not have an HRCT pattern of definite UIP, we suggest SLB (conditional recommendation, very low quality of evidence)	
**Added Recommendation**		**Source**
“Look for signs and symptoms (chronic dry cough, chest tightness, shortness of breath, mid to late inspiratory crackles, digital clubbing) that raise suspicion towards a diagnosis of IPF/ILD”		Medscape [32, 33]
“Assess risk factors – modifiable (environmental exposure of metal dust/wood particles, smoking history, exposure to pets) and non-modifiable (age over 50, male gender, family history) – for IPF”		Medscape [32, 34]
“Conduct rheumatological examination, along with general physical and respiratory examination in patients with suspicion of IPF.”		ATS/ERS [35, 36]
“For adult patients with suspicious signs and symptoms aligning with IPF, and a HRCT scan showing UIP pattern, refer to a pulmonologist or ILD clinic for specialized care.”		AAFP [37]
“For adult patients with suspicious signs and symptoms aligning with IPF, and a HRCT scan showing UIP pattern, recommend intervention for modifiable risk factors, if any. If comorbid GERD is present, start intervention.”		Medscape [32, 38]
“For adult patients with no suspicious signs and symptoms aligning with IPF, but with significant risk factors towards developing it, offer counselling and interventions for modifiable risk factors. If a patient is diagnosed with GERD, then start appropriate management.”		Medscape [32, 34]
“For adult patients with suspicious signs and symptoms aligning with IPF, HRCT scan showing UIP pattern, and patient hypoxemic (SpO2 < 88%), recommend home oxygen therapy to maintain saturation above 90% at rest, with sleep, and with exertion.”		Medscape [32, 33]

**Table 2:** Recommendations adopted with minor changes and those added to the clinical referral pathway for Primary Care Management of Bronchiectasis ABPA: Allergic Bronchopulmonary Aspergillosis. NIH: National Institutes of Health

Recommendations adopted with Minor Changes		
Original Recommendations	Recommendations Adopted with Minor Changes	
**“For P. aeruginosa colonised patients** Consider azithromycin or erythromycin as an alternative (eg, if a patient does not tolerate inhaled antibiotics) to an inhaled antibiotic for patients with bronchiectasis and chronic P. aeruginosa infection. (B)” [27]	**For P. aeruginosa colonised patients** Consider azithromycin as an alternative (e.g., if a patient does not tolerate inhaled antibiotics) to an inhaled antibiotic for patients with bronchiectasis and chronic *P. aeruginosa* infection. (B)	
**“For P. aeruginosa colonised patients** Consider azithromycin or erythromycin as an additive treatment to an inhaled antibiotic for patients with bronchiectasis and chronic P. aeruginosa infection who have a high exacerbation frequency. (D)” [27]	**For P. aeruginosa colonised patients** Consider azithromycin as an additive treatment to an inhaled antibiotic for patients with bronchiectasis and chronic *P. aeruginosa* infection who have a high exacerbation frequency. (D)	
**“For non-P. aeruginosa colonised patients** Use azithromycin or erythromycin for patients with bronchiectasis. (A)” [27]	**For non-P. aeruginosa colonised patients** Use azithromycin for patients with bronchiectasis. (A)	
**“For non-P. aeruginosa colonised patients** Consider inhaled gentamicin as a second line alternative to azithromycin or erythromycin. (B)” [27]	**For non-P. aeruginosa colonised patients** Consider inhaled gentamicin as a second line alternative to azithromycin. (B)	
**Added Recommendation**		**Source**
“Look for signs and symptoms (chronic productive cough with purulent or mucopurulent sputum, recurrent chest infections and shortness of breath) that raise suspicion towards a diagnosis of bronchiectasis”		Medscape [39, 40]
“Assess risk factors – modifiable (*smoking)* and non-modifiable (pre-existing conditions like cystic fibrosis, immunodeficiency disorders, connective tissue disorders, ABPA and recent severe pneumonia) – for bronchiectasis”		NIH National Heart, Lung and Blood Institute [41]
“For adult patients with no suspicious signs and symptoms aligning with bronchiectasis, but with significant risk factors towards developing it, offer counselling regarding the modifiable risk factors, in this case - smoking. Recommend cessation of smoking and avoiding inhaling secondhand smoke, if applicable.”		Medscape [39, 42]

**Table 3:** Recommendations added to the clinical referral pathway for Primary Care Management of Chronic Obstructive Pulmonary Disease ACP: American College of Physicians; ACCP: American College of Chest Physicians; ATS: American Thoracic Society; ERS: European Respiratory Society; COPD: Chronic Obstructive Pulmonary Disease; FEV1/FVC: Ratio of Forced Expiratory Volume in 1 s to Forced Vital Capacity of lungs; GOLD: Global Initiative for Chronic Obstructive Lung Disease

Added Recommendation	Source
“Look for signs and symptoms (persistent progressive dyspnea, chronic cough, sputum production) that raise suspicion towards a diagnosis of COPD”	Medscape [43] ACP, ACCP, ATS, ERS [44]
“Assess risk factors – modifiable (tobacco smoke exposure, smoke from home cooking, occupational irritants) and non-modifiable (family history, genetic factors) – for COPD”	Medscape [43, 45, 46]
“In individuals who have suspicious signs and symptoms aligning with COPD, and a FEV1/FVC ratio of > 70%, refer to specialist for further management.”	GOLD [47]
“In patients diagnosed with COPD, after recommendation of initial pharmacological and non-pharmacological therapies, call for follow-up in 2–4 weeks to review patients’ response to management.”	Expert Consensus

**Table 4:** Recommendations added to the clinical referral pathway for Primary Care Management of Asthma NICE: National Institute for Health and Care Excellence

Added Recommendation	Source
“Look for signs and symptoms (dry cough, shortness of breath, chest tightness, wheeze) that raise suspicion towards a diagnosis of asthma.”	Medscape [48, 49]
“Assess risk factors – modifiable (smoking, obesity, exposure to environmental irritants) and non-modifiable (personal/family history of atopy) – for asthma.”	Medscape (48, 50–52)
“In individuals who have suspicious signs and symptoms aligning with asthma, recommend spirometry, bronchodilator responsiveness testing and peak flow variability testing”	NICE [53] European Respiratory Society Guidelines for the Diagnosis of Asthma in Adults [54]
“In individuals who have suspicious signs and symptoms aligning with asthma, and upon diagnostic testing, have findings not suggestive of asthma, refer them to a specialist for further management.”	Medscape [49]

### Challenges and solutions

The challenges faced were broadly categorized into four main themes: resources, stakeholder support and involvement, resistance to change, and methodological limitations (Table 5).

**Table 5:** Challenges faced and proposed solutions EBCPG: Evidence-based Clinical Practice Guideline * Minor challenge; ** Major Challenge + The decision to limit widespread stakeholder involvement was to minimize undue delays stemming from factors including logistic difficulties, conflicts of interest, lack of mutual availability, political influences, and lack of direct incentives

Category of Challenge	Specific Challenge	Proposed Solution
**Resources**	• Inadequate expertise & experience with guideline development *: Our team was the first to implement GRADE-ADOLOPMENT of any guidelines in Pakistan in collaboration with the GRADE-USA working group.	**•** Collaborate with personnel with requisite experience and expertise **•** Conduct comprehensive, standardized training modules for all personnel involved in the “adolopment”
	• Inadequate manpower/size of workforce *: Our team consisted of 4–5 personnel, which limited pace of work.	**•** Involve students and trainees on a volunteer basis
**Stakeholder Support & Involvement**	• Suboptimal provincial/federal government involvement * ^+^: As we expected delays stemming from external stakeholder involvement, we decided to proceed without provincial/federal government involvement in this first iteration of GRADE-ADOLOPMENT. This may have led to possible public health and large-scale implementation perspectives missing from this effort.	• Involve all stakeholders from the start • Emphasize and reiterate mutual interests • Design specific curricula for all stakeholders involved • Tailor and deliver presentations to all stakeholders involved and invite stakeholders to participate in the GRADE-ADOLOPMENT process.
	• Suboptimal involvement of external societies or organizations * ^+^: As we expected delays and conflicts of interest stemming from involvement of external societies, we decided to proceed without their involvement in this first iteration of GRADE-ADOLOPMENT. This may lead to a possible future lack of buy-in from these organizations regarding the newly developed local EBCPGs.	
	• Absence of patients’ perspective in the “adolopment” process * ^+^: As we expected it nigh impossible to secure a patient sample sufficiently representative of the diverse sociodemographic and cultural population in Pakistan, we elected to consider the perspective of the experts as a surrogate for patients’. Nevertheless, this may lead to some socioeconomic and cultural considerations missing in our effort.	
**Resistance to Change**	• Rigorousness of the GRADE-ADOLOPMENT process may discourage the process of adaptation *: Given that the subject experts did not deem adaptation of any of the recommendations necessary, it is possible they were dissuaded from adaptation by the rigorousness of the process.	• Initial presentation to emphasize need for local EBCPGs, robustness of the GRADE-ADOLOPMENT process, and the importance of strict adherence to the rigorous GRADE-ADOLOPMENT processes in order to produce valid and credible guidelines.
**Methodological Limitations**	• Individual-level biases from experts **: Given unavoidable differences in each expert’s clinical practice, it is possible that individual-level biases affected the GRADE-ADOLOPMENT process.	• Increase the number and diversity of experts • Gauge acceptability and validity of any revisions made by getting feedback from experts from external institutes
	• Group-level biases from experts **: Given that all the experts were from a single institution, it is possible that group-level biases affected the GRADE-ADOLOPMENT process.	

## Discussion

We aimed to apply the GRADE-ADOLOPMENT process for EBCPGs creation for the primary care management of four chronic respiratory diseases, and create clinical pathways guiding diagnosis and referral, for general practitioners (GPs) in Pakistan. The primary care pathways derived from the created EBCPGs outlined steps for initial diagnosis by GPs and indications for specialist referral. The best-evidence review process resulted in the addition of seven recommendations for IPF, three recommendations for bronchiectasis, four recommendations for COPD, and four recommendations for asthma to cover gaps in practice.

In Pakistan, currently, there are no frameworks that guide GPs to manage patients with chronic respiratory conditions at the primary care level before referring them to a specialist. This results in patients either being referred without appropriate baseline investigations or not being diagnosed and referred at all, with the existing set-up is inefficient in its utilization of scare resources, time, funds, and limited specialist availability. The delays in specialist pulmonology care also stem from a low index of suspicion exercised by GPs and unsurety of how to proceed with diagnosis and referral [55]. Thus, the implementation of a standardized primary care triage framework may support the identification, appropriate work-up, and timely referral of patients suffering from potentially debilitating chronic lung diseases. The benefits of such systems are well-supported by experiences in other settings across the globe. In the United Kingdom, the implementation of dedicated proformas and care pathways at the primary care level results in considerable savings in healthcare costs due to reduced unwarranted specialist referrals [2]. Moreover, approximately 18 days are saved if GPs themselves refer patients for appropriate work-up, before specialist referral, as opposed to directly to a specialist without work-up [56]. In the Netherlands, the implementation of an asthma care pathway considerably improved GPs awareness regarding asthma care and improved their diagnostic and therapeutic approaches [57].

Amongst the most notable additions made were those pertaining to the management of IPF. This included a recommendation for home oxygen therapy for hypoxemic patients to maintain their SpO_2_ levels above 90% throughout the day, and the appropriate management of comorbid gastroesophageal reflux disease (GERD) in patients with IPF. The benefits of home oxygen therapy may range from improved exercise tolerance [58] and lesser dyspnea [58] to the attenuation of cardiac dysfunction [59]. Symptoms of GERD are present in more 33% of patients with IPF [38]. The relationship between GERD and IPF is reciprocal, with chronic reflux and micro aspiration causing alveolar injury and remodeling, and the increased negative intrathoracic pressure seen in IPF causing esophageal sphincter dysfunction [60]. Appropriate management of comorbid GERD can thus improve the prognosis of patients with IPF.

We faced several challenges in the creation of primary care pathways. Firstly, we were unable to find pre-existing EBCPGs that holistically covered all aspects of primary care management for the selected chronic respiratory conditions (diagnosis, basic management, and referral). Finer details, such as the signs and symptoms, and risk factors, for each disease were routinely missed. Thus, information had to be compiled from a variety of sources to develop a coherent and complete care pathway. Secondly, the recommendations within the selected source EBCPGs were not structured in a stepwise manner conducive to adaptation into a care pathway. In addition, it was difficult to establish what level of care could be expected from GPs at the primary care level, in terms of basic and sophisticated diagnostic modalities, and disease treatment. It was also important to remain cognizant of the considerable variability in available resources across primary care set-ups in different regions in Pakistan. The focus was thus majorly maintained on a focused history and examination, followed by basic diagnostic investigations, and prompt referral.

Our study has a few limitations. We did not include additional significant stakeholders, such as primary care physicians, nurses, patients, allied health professionals, experts outside of AKU, external organizations concerned with ENT, provincial or federal authorities. This decision was taken in order to minimize undue delays stemming from factors including logistical challenges, a lack of mutual availability, political pressures, conflict of interest, and a lack of incentives. These issues reflect hurdles to the application of the ideal GRADE-ADOLOPMENT process, particularly in LMICs like Pakistan, and exacerbate the processes’ intrinsic partiality. Moreover, while the benefits of primary care triage for chronic respiratory diseases in Pakistan are significant, the feasibility of such practice remains to be seen with particular concern for rural implementation. Rural locations in Pakistan often lack the infrastructure and healthcare facilities needed to provide specialist services, should patients require it. Geographical and financial barriers remain significant challenges to the implementation of the clinical diagnosis and referral pathways. Lastly, there is subjectivity in the TOR review process, as this is done by the individual experts, thus introducing possible bias in the decision to adopt, adapt, or create recommendations.

## Conclusion

Our study employed the GRADE-ADOLOPMENT process to create EPCPGs for four chronic respiratory conditions, namely IPF, bronchiectasis, asthma, and COPD. Furthermore, we also created clinical diagnosis and referral pathways for the primary care management of the aforementioned chronic respiratory conditions, clearly delineating a GPs role in diagnosis and timely referral. The widespread adoption of these EPCPGs and clinical pathways in the healthcare system of Pakistan can help alleviate the considerable burden of chronic respiratory illnesses in the country.

## Electronic supplementary material

Below is the link to the electronic supplementary material.

Supplementary Material 1

Supplementary Material 2

## Data Availability

All data generated or analyzed during this study are included in this published article and its supplementary information files.
