# Supplementary material for: GRADE-ADOLOPMENT of clinical practice guidelines and creation of clinical pathways for the primary care management of chronic respiratory conditions in Pakistan
Source: BMC Pulm Med. 2023 Apr 17;23:123. doi: 10.1186/s12890-023-02409-4 (PMC10111762; doi:10.1186/s12890-023-02409-4)
Supplement: Supplementary file 1 — Supplementary Material 1 [file 12890_2023_2409_MOESM1_ESM.docx]

**Supplement**

1. *GRADE-ADOLOPMENT process for adaptation using Evidence to Decision (EtD) tables.*
2. *Supplement Section 1: Criteria evaluated in the Evidence to Decision (EtD) tables.*
3. *Supplement Section 2: Dummy Evidence to Decision (EtD) table.*
4. *Excluded recommendations (Supplement Section 3-6).*
5. *Table of recommendations for diagnosis and management of asthma, chronic obstructive pulmonary disease, idiopathic pulmonary fibrosis, and bronchiectasis.*

**GRADEPro Evidence to Decision Framework**

GRADEPro is a web application used to help create, manage, and share summaries of research evidence (1). The CCBP staff involved in this study underwent a training module to master use of GRADEPro for the GRADE-ADOLOPMENT process. The software will be used to develop Evidence to Decision (EtD) tables to reach a consensus on any recommendation marked as “*Adapt*.”

EtD tables (1) are tables that summarize evidence to enable members of an expert panel to make healthcare recommendations or decisions. Development of EtD tables begins with formulation of a question structured as follows: “Should the *Intervention/Suggested Change* be favored over the *Comparison/Current Standard of Practice?*” The pros and cons of the suggested change are judged by an expert panel across 12 criteria, that are shown in **Supplement Section 1**.

Each criterion will be supported with evidence gathered through a best evidence review process, to provide local context for the pros and cons of the recommendation. The CCBP team will summarize the newly gathered evidence for each criterion in the “*Research Evidence*” and “*Additional Considerations*” columns.

**Best-Evidence Review**

A best-evidence review was conducted to source research evidence that would help a recommendation be assessed across all 12 criteria. The best-evidence review was conducted separately for each of the 12 criteria and included a mini-systematic review and review of supporting evidence.

- *Mini-Systematic Review*: A mini-systematic review follows the same general protocol as a full systematic review, but applies arbitrary selection criteria (such as geographical region of publication) or limits the number of databases searched (2). In our mini-systematic review, PubMed and Google Scholar were queried using a search string designed using keywords from the recommendation in question. To maintain a local focus, only articles reporting data relevant to Pakistan were selected.

Two members of CCBP staff independently screened the titles and abstracts of articles sourced from PubMed and Google, with only those reporting relevant information specific to Pakistan undergoing a full-text review to finalize inclusion. As the source guideline itself was produced based on a systematic review process, careful full-text review of the bibliography within the source document was conducted. Two members of the CCBP staff extracted appropriate evidence from the final list of articles included.

- *Supporting Evidence*: Information pertaining to the cost of different investigations and treatments, as well as the availability of diagnostic and management facilities, was sourced from a selection of local hospitals, healthcare facilities, and pharmacies via telephonic query and their websites.

**Expert Panel Review**

An expert panel of 5-15 faculty members from AKU will be invited by the Section Head to review the completed EtD table for each recommendation and provide their judgement for each criterion. This judgment is in the form of a single selection from multiple response options. If, for any criteria, an expert requires additional evidence, they will be instructed to inform the CCBP team. Effort will be made to source the requisite information, which, if found, is shared with all the panel members. Experts’ judgement will be sought in an anonymous and confidential manner, with the GRADEPro software allowing reviewers to select options and provide feedback without their identity known to fellow experts. A sample of a GRADEPro EtD is shown as **Supplement Section 2**.

**Final Recommendation Revisions & Synthesis**

Once all the members of the expert panel have provided their responses to the EtD, the CCBP staff synthesizes responses to produce a summary of judgments. The CCBP staff then conducts a meeting with the expert panel to review the summary of judgments and reach a final unanimous consensus on the need for and nature of any revisions to the recommendations in question. The strength of each recommendation is also decided. Finally, the consensus is presented to the Section Head for review, after which the recommendation is incorporated into the Pakistani EBCPG with a summary of the consensus decision.

| **Supplement Section 1: Criteria evaluated in the Evidence to Decision (EtD) tables** | | |
| --- | --- | --- |
| **Criteria** | **Description** | **Interpretation** |
| **Problem** | The magnitude of a problem, as measured by its prevalence and severity in a local context | The more serious or urgent a problem is, the more likely that the option that better addresses the problem receives a strong recommendation. |
| **Desirable Effects** | The magnitude of desirable effects is judged by considering the importance of the outcome and the size of the desirable effects (likelihood of experiencing a benefit or degree of benefits an individual experiences). | An option with greater desirable effects is more likely to gain a strong recommendation. |
| **Undesirable Effects** | The magnitude of undesirable effects is similarly judged by considering the importance of the outcome and the size of the undesirable effects. | An option with fewer undesirable effects is more likely to gain a strong recommendation. |
| **Certainty of Evidence** | Determined by likelihood that the research provides valid evidence regarding the effect of the option on all critical outcomes. | Evidence with higher certainty lends to a strong recommendation. |
| **Values** | Magnitude of value is judged by the variability or uncertainty of weightage placed upon the outcome by individuals. | Less variability/uncertainty of value leads to a strong recommendation. |
| **Balance of Effects** | The balance of effects is judged by considering the value individuals place upon the main outcomes, the degree of desirable and undesirable effects, and the certainty of those estimates. | The overall balance of effects can be judged as either favoring the intervention or comparison. |
| **Resources Required** | An estimate of the cost of the difference in resource use between the intervention and comparison. | An option with large savings is more likely to receive a strong recommendation. |
| **Certainty of Evidence of Required Resources** | It is determined by the likelihood that the research provides valid evidence of cost differences between the intervention and comparison. | Evidence with higher certainty lends to a strong recommendation. |
| **Cost-Effectiveness** | Determines cost-effectiveness by considering uncertainty about or variability in costs or net benefit, sensitivity analyses, and the reliability and applicability of the economic evaluation. | An option that is more cost-effective is more likely to receive a strong recommendation |
| **Equity** | Likelihood of differences in the relative effectiveness of the intervention for disadvantaged subgroups that influence the absolute effectiveness of the intervention. | An option with a greater likelihood to favor equity is more likely to receive a strong recommendation. |
| **Acceptability** | Likelihood of key stakeholders to accept the distribution of benefits, harms, costs, and ethical concerns associated with the intervention, over an extended period. | An option more acceptable to most stakeholders is more likely to receive a strong recommendation. |
| **Feasibility** | Practicality of sustained use of the intervention. | An option more feasible to most stakeholders is more likely to receive a strong recommendation. |

| **Supplement Section 2: Dummy Evidence to Decision (EtD) table** | | | |
| --- | --- | --- | --- |
| **Question**: Should *Intervention/Suggested Change* be favored over *Comparison/Current Standard of Practice*? | | | |
| **Criteria** | **Research Evidence** | **Additional Considerations** | **Judgment** |
| **Problem:** Is the problem a priority? |  |  | - No - Probably No - Probably Yes - Yes - Varies - Don’t Know |
| **Desirable Effects:** How substantial are the desirable anticipated effects? |  |  | - Trivial - Small - Moderate - Large - Varies - Don’t Know |
| **Undesirable Effects**: How substantial are the undesirable anticipated effects? |  |  | - Large - Moderate - Small - Trivial - Varies - Don’t Know |
| **Certainty of Evidence**: What is the overall certainty of the evidence of effects? |  |  | - Very Low - Low - Moderate - High - No Included Studies |
| **Value**: Is there important uncertainty about or variability in how much people value the main outcomes? |  |  | - Important Uncertainty or Variability - Possible Uncertainty or Variability - Probably No Important Uncertainty or Variability - No Important Variability or Uncertainty |
| **Balance of Effects**: Does the balance between desirable and undesirable effects favor the intervention or the comparison? |  |  | - Favors Comparison - Probably Favors the Comparison - Does Not Favor Either the Intervention or Comparison - Probably Favors the Intervention - Favors Intervention - Varies - Don’t Know |
| **Resources Required**: How large are the resource requirements (costs)? |  |  | - Large Costs - Moderate Costs - Negligible Costs or Savings - Moderate Savings - Large Savings - Varies - Don’t Know |
| **Certainty of Evidence of Required Resources**: What is the certainty of the evidence of resource requirements (costs)? |  |  | - Very Low - Low - Moderate - High - No Included Studies |
| **Cost-Effectiveness**: Does the cost-effectiveness of the intervention favor the intervention or the comparison? |  |  | - Favors Comparison - Probably Favors the Comparison - Does Not Favor Either the Intervention or Comparison - Probably Favors the Intervention - Favors Intervention - Varies - No Included Studies |
| **Equity**: What would be the impact on health equity? |  |  | - Reduced - Probably Reduced - Probably No Impact - Probably Increased - Increased - Varies - Don’t Know |
| **Acceptability**: Is the intervention acceptable to key stakeholders? |  |  | - No - Probably No - Probably Yes - Yes - Varies - Don’t Know |
| **Feasibility**: Is the intervention feasible to implement? |  |  | - No - Probably No - Probably Yes - Yes - Varies - Don’t Know |
| **Overall Recommendations**   - Strong Recommendation Against the Intervention - Conditional Recommendation Against the Intervention - Conditional Recommendation for Either the Intervention or the Comparison - Conditional Recommendation for the Intervention - Strong Recommendation for the Intervention | | | |

**Exclusions**

**Idiopathic Pulmonary Fibrosis**

| **Supplement Section 3: Exclusions made in the *EBCPG for IPF.***  Out of a total of 24 recommendations, 2 were excluded. | | |
| --- | --- | --- |
| **S.No** | **Recommendation** | **Reason for Exclusion** |
| 1 | For patients with newly detected ILD of apparently unknown cause who are clinically suspected of having IPF and have an HRCT pattern of probable UIP, indeterminate for UIP, or an alternative diagnosis, the panel made no recommendation for or against TBBx. | Beyond scope of a General Physician. |
| 2 | For patients with newly detected ILD of apparently unknown cause who are clinically suspected of having IPF and have an HRCT pattern of probable UIP, indeterminate for UIP, or an alternative diagnosis, the panel made no recommendation regarding lung cryobiopsy. | Unavailable in Pakistan. |

**Bronchiectasis**

| **Supplement Section 4: Exclusions made in the *EBCPG for Bronchiectasis.***  Out of a total of 59 recommendations, 1 was excluded. | | |
| --- | --- | --- |
| **S.No** | **Recommendations** | **Reason for Exclusion** |
| 1 | Consider measuring baseline specific antibody levels against capsular polysaccharides of *Streptococcus pneumoniae* in all patients to investigate for specific antibody deficiency. If pneumococcal antibodies are low, immunize with 23 valent polysaccharide pneumococcal vaccine, followed by measurement of specific antibody levels 4–8 weeks later. (D) | Unavailable in Pakistan. |

**Chronic Obstructive Pulmonary Disease**

| **Supplement Section 5: Exclusions made in the *EBCPG for Chronic Obstructive Pulmonary Disease (COPD)***  Out of a total of 93 recommendations, 12 were excluded. | | |
| --- | --- | --- |
| **S.No** | **Recommendations** | **Reason for Exclusion** |
| 1 | Simvastatin does not prevent exacerbations in COPD patients at increased risk of exacerbations and without indications for statin therapy | Not practiced in Pakistan. |
| 2 | Intravenous augmentation therapy may slow down the progression of emphysema. | Unavailable in Pakistan. |
| 3 | Alpha-1 antitrypsin augmentation therapy:  The logical approach to minimize the development and progression of lung disease in AATD patients is alpha-1-antitrypsin augmentation. Such therapy has been available in many, though not all, countries since the 1980s. Because AATD is rare, formal clinical trials to assess efficacy with conventional spirometric outcome have never been undertaken. However, a wealth of observational studies suggests a reduction in spirometric progression in treated versus non-treated patients and that this reduction is most effective for patients with FEV1 35-49% predicted. Never or ex-smokers with an FEV1 of 35-60% predicted have been suggested as those most suitable for AATD augmentation therapy | Unavailable in Pakistan. |
| 4 | Integrative care and telehealth have no demonstrated benefit at this time. | Unavailable in Pakistan. |
| 5 | Statin therapy is not recommended for prevention of exacerbations. | Recurring recommendation |
| 6 | Patients with hereditary alpha-1 antitrypsin deficiency and established emphysema may be candidates for alpha-1 antitrypsin augmentation therapy. | Unavailable in Pakistan. |
| 7 | Antitussives cannot be recommended. | Recurring recommendation |
| 8 | Resting oxygenation at sea level does not exclude the development of severe hypoxemia when traveling by air. | Recurring recommendation |
| 9 | In select patients with advanced emphysema, bronchoscopic interventions reduce end-expiratory lung volume and improve exercise tolerance, quality of life and lung function at 6-12 months following treatment: Endobronchial valve | Recurring recommendation |
| 10 | In select patients with advanced emphysema, bronchoscopic interventions reduce end-expiratory lung volume and improve exercise tolerance, quality of life and lung function at 6-12 months following treatment: Lung coils | Recurring recommendation |
| 11 | In select patients with advanced emphysema, bronchoscopic interventions reduce end-expiratory lung volume and improve exercise tolerance, quality of life and lung function at 6-12 months following treatment: Vapor ablation | Recurring recommendation |
| 12 | Short-acting inhaled beta_2_-antagonists with or without short-acting anticholinergics, are recommended as the initial bronchodilators to treat an acute exacerbation. | Not part of outpatient management |

**Asthma**

| **Supplement Section 6: Exclusions made in the *EBCPG for Asthma.***  Out of a total of 102 recommendations, 31 were excluded. | | |
| --- | --- | --- |
| **S.No** | **Recommendation** | **Reason for Exclusion** |
| 1 | Lung function should also be recorded more frequently in children based on asthma severity and clinical course (Evidence D). | Pediatric recommendation |
| 2 | In children, FeNO-guided treatment significantly reduces exacerbation rates compared with guidelines-based treatment (Evidence A)[.](#_bookmark359) | Pediatric recommendation |
| 3 | **Step 1 treatment options for children 6–11 years**  Possible controller options for this age-group include taking ICS whenever SABA is taken (Evidence B).  Regular ICS with as-needed SABA is also a possible option for this age-group (Evidence B). | Pediatric recommendation |
| 4 | **Preferred Step 3 treatment for children 6–11 years**  In children, after checking inhaler technique and adherence, and treating modifiable risk factors, there are three preferred options at a population level: to increase ICS to medium dose (Evidence A) or change to combination low dose ICS-LABA (Evidence A)[,](#_bookmark417) both with as-needed SABA reliever, or to switch to maintenance and reliever therapy with a very low dose of ICS-formoterol (Evidence B). | Pediatric recommendation |
| 5 | **Preferred Step 4 treatment for children 6–11 years**  For children whose asthma is not adequately controlled by low dose maintenance ICS-LABA with as-needed SABA, treatment may be increased to medium dose ICS-LABA (Evidence B).  For maintenance and reliever therapy with budesonide-formoterol, the maintenance dose may be increased to 100/6 mcg twice daily (metered dose; 80/4.5 mcg delivered dose) (Evidence D). | Pediatric recommendation |
| 6 | **Other Step 4 options for children 6–11 years**  Tiotropium (long-acting muscarinic antagonist) by mist inhaler may be used as add-on therapy in children aged 6 years and older; it modestly improves lung function and reduces exacerbations (Evidence A) largely independent of baseline IgE or blood eosinophils. | Pediatric recommendation |
| 7 | The benefit of this regimen (**Combination low dose ICS (budesonide or beclomethasone) with formoterol maintenance and reliever regimen)** in preventing exacerbations appears to be due to intervention at a very early stage of worsening asthma. This regimen was also effective in reducing exacerbations in children aged 4–11 years (Evidence B). | Pediatric recommendation |
| 8 | For children 6–11 years, the recommended dose of prednisone is 1–2 mg/kg/day to a maximum of 40 mg/day (Evidence B). | Pediatric recommendation |
| 9 | A trial of treatment for at least 2–3 months with as-needed short-acting beta_2_-agonist (SABA) and regular low dose inhaled corticosteroids (ICS) may provide some guidance about the diagnosis of asthma (Evidence D). | Pediatric recommendation |
| 10 | **Assessment of asthma** In young children, as in older patients, both symptom control and future risk should be monitored (Evidence D). **Assessing asthma symptom control** It incorporates assessment of symptoms; the child’s level of activity and their need for reliever/rescue treatment; and assessment of risk factors for adverse outcomes (Evidence D). | Pediatric recommendation |
| 11 | **Which children should be prescribed regular controller treatment?**  If the history and symptom pattern suggest a diagnosis of asthma (Box 6-2) and respiratory symptoms are uncontrolled and/or wheezing episodes are frequent (e.g., three or more episodes in a season), regular controller treatment should be initiated (Step 2) and the response evaluated (Evidence D).   - Regular controller treatment may also be indicated in a child with less frequent, but more severe episodes of viral- induced wheeze (Evidence D). - If the diagnosis of asthma is in doubt, and inhaled SABA therapy or courses of antibiotics need to be repeated frequently, e.g., more than every 6–8 weeks, a trial of regular controller treatment should be considered to confirm whether the symptoms are due to asthma (Evidence D). | Pediatric recommendation |
| 12 | **STEP 1: As-needed inhaled short-acting beta2-agonist (SABA)**  **Preferred option: as-needed inhaled short-acting beta_2_-agonist (SABA)**  All children who experience wheezing episodes should be provided with inhaled SABA for relief of symptoms (Evidence D).  **Other options**  Oral bronchodilator therapy is not recommended due to its slower onset of action and higher rate of side-effects compared with inhaled SABA (Evidence D). | Pediatric recommendation |
| 13 | **STEP 2: Initial controller treatment plus as-needed SABA**  **Preferred option: regular daily low dose ICS plus as-needed SABA**  Regular daily, low dose ICS is recommended as the preferred initial treatment to control asthma in children 5 years and younger (Evidence A).  **Other options**  In young children with persistent asthma, regular treatment with a leukotriene receptor antagonist (LTRA) modestly reduces symptoms and need for oral corticosteroids compared with placebo. However, for young children with recurrent viral- induced wheezing, a recent review concluded that neither regular nor intermittent LTRA reduces OCS-requiring exacerbations (Evidence A). | Pediatric recommendation |
| 14 | **STEP 3: Additional controller treatment, plus as-needed SABA and consider specialist referral.**  **Preferred option: medium dose ICS (double the ‘low’ daily dose)**  Doubling the initial low dose of ICS may be the best option (Evidence C).  **Other options**  Addition of a LTRA to low dose ICS may be considered, based on data from older children (Evidence D). | Pediatric recommendation |
| 15 | **STEP 4: Continue controller treatment and refer for expert assessment**  **Preferred option: refer the child for expert advice and further investigation (Evidence D).**  **Other options**  Further increase the dose of ICS for a few weeks until the control of the child’s asthma improves (Evidence D).  Add LTRA (data based on studies in older children, Evidence D).   - Add a low dose of oral corticosteroid (for a few weeks only) until asthma control improves (Evidence D). - Add intermittent high dose ICS at onset of respiratory illnesses to the regular daily ICS if exacerbations are the main problem (Evidence D). | Pediatric recommendation |
| 16 | **Reviewing response and adjusting treatment** Asthma-like symptoms remit in a substantial proportion of children of 5 years or younger so the need for continued controller treatment should be regularly assessed (e.g. every 3–6 months) (Evidence D).  If therapy is stepped-down or discontinued, schedule a follow-up visit 3–6 weeks later to check whether symptoms have recurred, as therapy may need to be stepped-up or reinstituted (Evidence D). | Pediatric recommendation |
| 17 | **Choice of Inhaler Device** Inhaled therapy constitutes the cornerstone of asthma treatment in children 5 years and younger. A pressurized metered-dose inhaler (pMDI) with a valved spacer (with or without a face mask, depending on the child’s age) is the preferred delivery system (Evidence A). | Pediatric recommendation |
| 18 | **Asthma Self-Management Education for Carers of Young Children**  Crucial to a successful asthma education program are a partnership between patient/carer and health care providers, with a high level of agreement regarding the goals of treatment for the child, and intensive follow-up (Evidence D). | Pediatric recommendation |
| 19 | **Written asthma action plans** A written action plan should be provided for the family/carers of all children with asthma, including those aged 5 years and younger (Evidence D). | Pediatric recommendation |
| 20 | **Initial home management of asthma exacerbations** Initial management includes an action plan to enable the child’s family members and carers to recognize worsening asthma and initiate treatment, recognize when it is severe, identify when urgent hospital treatment is necessary, and provide recommendations for follow up (Evidence D). | Pediatric recommendation |
| 21 | **Initial treatment at home** **Inhaled SABA via a mask or spacer, and review response**  The parent/carer should initiate treatment with two puffs of inhaled SABA (200 mcg salbutamol or equivalent), given one puff at a time via a spacer device with or without a facemask (Evidence D). | Pediatric recommendation |
| 22 | **Primary care or hospital management of acute asthma exacerbations in children 5 years or younger**  **Assessment of exacerbation severity**  Conduct a brief history and examination concurrently with the initiation of therapy. The presence of any of the features of a severe exacerbation are an indication of the need for urgent treatment and immediate transfer to hospital (Evidence D). | Pediatric recommendation |
| 23 | **Indications for immediate transfer to hospital** Children with features of a severe exacerbation that fail to resolve within 1–2 hours despite repeated dosing with inhaled SABA must be referred to hospital for observation and further treatment (Evidence D). | Pediatric recommendation |
| 24 | **Emergency treatment and initial pharmacotherapy** **Oxygen**  Treat hypoxemia urgently with oxygen by face mask to achieve and maintain percutaneous oxygen saturation 94–98% (Evidence A). | Pediatric recommendation |
| 25 | **Bronchodilator therapy**  The initial dose of SABA may be given by a pMDI with spacer and mask or mouthpiece or an air-driven nebulizer; or, if oxygen saturation is low, by an oxygen-driven nebulizer. For most children, pMDI plus spacer is favored as it is more efficient than a nebulizer for bronchodilator delivery (Evidence A). | Pediatric recommendation |
| 26 | **Assessment of response and additional bronchodilator treatment** Consider adding 1–2 puffs of ipratropium. Failure to respond at 1 hour, or earlier deterioration, should prompt urgent admission to hospital, addition of nebulized ipratropium, and a short course of oral corticosteroids (Evidence D).  Children who fail to respond to 10 puffs of inhaled SABA within a 3–4-hour period should be referred immediately to hospital (Evidence D).  If symptoms resolve rapidly after initial bronchodilator and do not recur for 1–2 hours: no further treatment may be required. Further SABA may be given every 3–4 hours (up to a total of 10 puffs/24 hours) and, if symptoms persist beyond 1-day, other treatments including inhaled and/or oral corticosteroids are indicated (Evidence D).  If inhalation is not possible an intravenous bolus of terbutaline 2 mcg/kg may be given over 5 minutes, followed by continuous infusion of 5 mcg/kg/hour(Evidence C). | Pediatric recommendation |
| 27 | **Maintain current controller treatment (if prescribed)**  Children who have been prescribed maintenance therapy with ICS, LTRA or both should continue to take the prescribed dose during and after an exacerbation (Evidence D). | Pediatric recommendation |
| 28 | **Inhaled corticosteroids**  For children not previously on ICS, an initial dose of ICS twice the low daily dose may be given and continued for a few weeks or months (Evidence D).  For those children already on ICS, doubling the dose was not effective in a small study of mild-moderate exacerbations in children aged 6–14 years, nor was quintupling the dose in children aged 5–11 years with good adherence. This approach should be reserved mainly for individual cases and should always involve regular follow up and monitoring of adverse effects (Evidence D). | Pediatric recommendation |
| 29 | **Oral corticosteroids**  For children with severe exacerbations, a dose of OCS equivalent to prednisolone 1–2 mg/kg/day, with a maximum of 20 mg/day for children under 2 years of age and 30 mg/day for children aged 2–5 years, is currently recommended (Evidence A[)](#_bookmark866).  Although several studies have failed to show any benefits when given earlier (e.g., by parents) during periods of worsening wheeze managed in an outpatient setting (Evidence D).  A course of 3–5 days is sufficient in most children of this age and can be stopped without tapering (Evidence D). | Pediatric recommendation |
| 30 | **Discharge and follow up after an exacerbation.** Children who have recently had asthma exacerbation are at risk of further exacerbations and require follow up. The purpose is to ensure complete recovery, to establish the cause of the exacerbation, and, when necessary, to establish appropriate maintenance treatment and adherence (Evidence D).  Prior to discharge from the emergency department or hospital, family/carers should receive the following advice and information (all are Evidence D). | Pediatric recommendation |
| 31 | **Primary prevention of asthma****Factors associated with increased or decreased risk of asthma in children.** Breastfeeding  Breastfeeding decreases wheezing episodes in early life; however, it may not prevent development of persistent asthma (Evidence D).  Regardless of its effect on development of asthma, breastfeeding should be encouraged for all of its other positive benefits (Evidence A). | Pediatric recommendation |

**DIAGNOSIS AND MANAGEMENT OF ASTHMA**

**Source Guideline**: Global strategy for asthma management and prevention (2021 update)

**Key to understanding level of evidence and strength of recommendation.**

| Level of Evidence | | | |
| --- | --- | --- | --- |
| Evidence Level A | Evidence Level B | Evidence Level C | Evidence Level D |
| Randomized controlled trials (RCTs), systematic reviews, observational evidence. Rich body of data. | Randomized controlled trials (RCTs) and systematic reviews. Limited body of data. | Nonrandomized trials or observational studies. | Panel consensus judgement. |

**Table of Recommendations**

|  | Assessment of asthma |
| --- | --- |
|  | Assess lung function at diagnosis or start of treatment; after 3–6 months of controller treatment to assess the patient’s personal best FEV1; and periodically thereafter. In most adult patients, record lung function at least every 1-2 years, but more frequently in higher risk patients including those with exacerbations and those at risk of decline in lung function.  [Evidence D] |
|  | Self-management education reduces asthma morbidity in adults.  [Evidence A] |
|  | Good communication by health care providers is essential as basis for good outcomes.  [Evidence B] |
|  | Treatment guided by fractional concentration of exhaled nitric oxide (FeNO) |
|  | In young adults, FeNO-guided treatment is associated with a significant reduction in number of patients with ≥1 exacerbation and in exacerbation rate.  [Evidence A] |
|  | Offer sputum-guided treatment for adult patients with moderate or severe asthma who are managed in (or can be referred to) centres experienced in this technique  [Evidence A] |
|  | Preferred Step 1 treatment: (Track 1) |
|  | Use of low dose ICS-formoterol as needed for symptom relief in Step 1 for adults and adolescents. [Evidence B] |
|  | Patients with mild asthma who are prescribed as-needed ICS-formoterol to prevent exacerbations and control symptoms can use the same medication prior to exercise, if needed, and do not need to be prescribed a SABA for pre-exercise use  [Evidence B] |
|  | Alternative Step 1 (Track 2) |
|  | Low dose ICS taken whenever SABA is taken.  [Evidence B] |
|  | Avoid SABA-only treatment in adults or adolescents. Patients whose asthma is treated with SABA alone (compared with ICS) are at increased risk of asthma-related death.  [Evidence A]  and urgent asthma-related healthcare.  [Evidence A] |
|  | Use of regular or frequent LABA without ICS is strongly discouraged because of risk of exacerbations.  [Evidence A] |
|  | Preferred Step 2 treatment (Track 1) |
|  | Offer low dose ICS-formoterol, taken as-needed for relief of symptoms.  [Evidence A] |
|  | In patients with mild asthma use of as-needed ICS-formoterol, as-needed budesonide-formoterol is superior to maintenance ICS in reducing the risk of severe exacerbations.  [Evidence A] |
|  | Patients with mild asthma who are prescribed as- needed ICS-formoterol to prevent exacerbations and control symptoms can use the same medication prior to exercise, if needed, and do not need to be prescribed a SABA for pre-exercise use.  [Evidence B] |
|  | Alternative Step 2 (Track 2) |
|  | Offer daily low dose ICS plus as-needed SABA, risks of severe exacerbations, hospitalizations and mortality are substantially reduced with regular low dose ICS; symptoms and exercise-induced bronchoconstriction are also reduced.  [Evidence A] |
|  | Other Step 2 options |
|  | Leukotriene receptor antagonists (LTRA) are less effective than ICS[,](#_bookmark393) particularly for exacerbations. [Evidence A] |
|  | In patients not previously using controller treatment, regular daily combination low dose ICS-LABA as initial maintenance controller treatment reduces symptoms and improves lung function compared with low dose ICS alone. However, it is more expensive and does not further reduce risk of exacerbations compared with ICS alone.  [Evidence A] |
|  | For patients with purely seasonal allergic asthma, e.g., with birch pollen, with no interval asthma symptoms, regular daily ICS or as-needed ICS-formoterol should be started immediately once symptoms commence and be continued for four weeks after pollen season ends.  [Evidence D] |
|  | Do not offer sustained-release theophylline as it has only weak efficacy in asthma.  [Evidence B] |
|  | Avoid chromones (nedocromil sodium and sodium cromoglycate), as they have a favorable safety profile but low efficacy.  [Evidence A] |
|  | Preferred Step 3 (Track 1) |
|  | In patients with ≥1 exacerbation in the previous year, offer low dose ICS-formoterol maintenance and reliever therapy.  [Evidence A] |
|  | Alternative Step 3 (Track 2) |
|  | Offer maintenance ICS-LABA plus as-needed SABA.  [Evidence A] |
|  | Other Step 3 options |
|  | Increase ICS to medium dose, but at a group level this is less effective than adding a LABA.  [Evidence A] |
|  | Other less efficacious options are low dose ICS plus either LTRA  [Evidence A]  or low dose, sustained-release theophylline.  [Evidence B] |
|  | Preferred Step 4 (Track 1) |
|  | Offer medium dose ICS-formoterol maintenance and reliever therapy.  [Evidence A] |
|  | Alternative Step 4 (Track 2) |
|  | Offer medium or high dose ICS-LABA with as-needed SABA, if maintenance and reliever therapy is not available.  [Evidence B] |
|  | Other Step 4 options |
|  | Add LAMA to medium or high dose ICS-LABA.  [Evidence A] |
|  | Offer medium or high dose budesonide, dosing four times daily.  [Evidence B] |
|  | For other ICS, twice-daily dosing is appropriate.  [Evidence D] |
|  | Other options include LTRA  [Evidence A]  or low dose sustained-release theophylline  [Evidence B] |
|  | Preferred treatment at Step 5 |
| Refer  to  Specialist | Refer patients of any age with persistent symptoms or exacerbations despite correct inhaler technique and good adherence with Step 4 treatment and in whom other controller options have been considered, to a specialist with expertise in investigation and management of severe asthma.  [Evidence D] |
|  | - Consider combination high dose ICS-LABA. - [Evidence A] |
|  | - Use only on a trial basis for 3–6 months when good asthma control cannot be achieved with medium dose ICS plus LABA and/or a third controller (e.g., LTRA or sustained-release theophylline. - [Evidence B] |
|  | - Add-on long-acting muscarinic antagonists (LAMA) as these modestly improve lung function, - [Evidence A] but not symptoms. |
|  | - Add-on LAMA modestly increases time to severe exacerbation requiring oral corticosteroids. - [Evidence B] |
| Refer  to  Specialist | - Consider add-on azithromycin (three times a week) after specialist referral for adult patients with persistent symptomatic asthma despite high dose ICS-LABA. - [Evidence B] |
|  | - Add-on anti-immunoglobulin E (anti-IgE) (Omalizumab) treatment for patients aged ≥6 years with moderate or severe allergic asthma that is uncontrolled on Step 4–5 treatment. - [Evidence A] |
|  | - Add-on anti-interleukin-5/5R treatment; intravenous reslizumab for ages ≥18 years or subcutaneous benralizumab for ages ≥12 years, with severe eosinophilic asthma that is uncontrolled on Step 4–5 treatment. - [Evidence A] |
|  | - Add-on anti-interleukin-4R treatment (subcutaneous dupilumab) for patients aged ≥12 years with severe Type 2 asthma or requiring treatment with maintenance OCS. - [Evidence A] |
|  | - Sputum-guided treatment |
|  | - For adults with persisting symptoms and/or exacerbations despite high dose ICS or ICS-LABA, adjust treatment based on eosinophilia (>3%) in induced sputum. In severe asthma, this leads to reduced exacerbations and/or lower doses of ICS. - [Evidence A] |
|  | - Consider add-on treatment with bronchial thermoplasty for adult patients with severe asthma. - [Evidence B] |
|  | - Add-on low dose oral corticosteroids (≤7.5 mg/day prednisone equivalent) in adults with severe asthma. [Evidence D] - but these are often associated with substantial side effects. - [Evidence A] |
|  | Review of Asthma |
|  | Frequency of visits depends upon patient’s initial level of control, their response to treatment, their level of engagement in self-management. Ideally, patients should be seen 1–3 months after starting treatment and every 3–12 months thereafter. Schedule a review visit within 1 week after an exacerbation.  [Evidence D] |
|  | Step down treatment |
|  | Do not step-down treatment too far or too quickly, as exacerbation risk may increase even if symptoms remain reasonably controlled.  [Evidence B] |
|  | Avoid complete cessation of ICS as it is associated with a significantly increased risk of exacerbations.  [Evidence A] |
|  | General principles of stepping down treatment |
|  | - Consider stepping down when asthma symptoms have been well controlled and lung function has been stable for 3 or more months. - [Evidence D] |
|  | Engage patient in the process; document their asthma status (symptom control, lung function and risk factors); provide clear instructions; provide a written asthma action plan and ensure patient has sufficient medication to resume their previous dose if necessary; monitor symptoms and/or PEF; and schedule a follow-up visit.  [Evidence D] |
|  | Stepping down ICS doses by 25–50% at 3-month intervals is feasible and safe for most patients. [Evidence A] |
|  | Subcutaneous immunotherapy (SCIT) |
|  | Weigh potential benefits of SCIT against risk of adverse effects and inconvenience and cost of prolonged course of therapy, including the minimum half-hour wait required after each injection.  [Evidence D] |
|  | Sublingual immunotherapy (SLIT) |
|  | Consider adding SLIT for adult patients with allergic rhinitis and sensitized to house dust mite, with persisting asthma symptoms despite low-medium dose ICS-containing therapy, provided FEV1 is >70% predicted.  [Evidence B] |
|  | Vaccinations |
|  | Advise patients with moderate to severe asthma to receive an influenza vaccination every year, or at least when vaccination of the general population is advised.  [Evidence C] |
|  | There is insufficient evidence to recommend routine pneumococcal vaccination in people with asthma.[Evidence D] |
|  | Smoking cessation and avoidance of environmental tobacco smoke |
|  | Strongly encourage people with asthma who smoke to quit. Provide them with access to counselling and, if available, to smoking cessation programs.  [Evidence A] |
|  | Strongly encourage patients to avoid environmental smoke exposure.[Evidence B] |
|  | Physical activity |
|  | Encourage patients to engage in regular physical activity because of its general health benefits.  [Evidence A] |
|  | Provide patients with advice about prevention and management of exercise-induced bronchoconstriction including with daily treatment with ICS[Evidence A]plus SABA as-needed and pre-exercise[Evidence A]or with low dose ICS-formoterol as-needed and before exercise[Evidence B]with warm-up before exercise if needed[Evidence A] |
|  | Avoidance of occupational exposures |
|  | Identify and eliminate occupational sensitizers as soon as possible and remove sensitized patients from any further exposure to these agents.  [Evidence A] |
| Refer  to  Specialist | Patients with suspected or confirmed occupational asthma should be referred for expert assessment and advice, if available, because of economic and legal implications of diagnosis.  [Evidence A] |
|  | Avoidance of medications that may make asthma worse |
|  | Always ask about concomitant medications, including eyedrops.  [Evidence D]   - Aspirin and NSAIDs are not generally contraindicated in asthma unless there is a history of previous reactions to these agents. - [Evidence A] |
|  | Asthma should not be regarded as an absolute contraindication to use cardio-selective beta-blockers when they are indicated for acute coronary events, but the relative risks and benefits should be considered.[Evidence D] |
|  | Avoidance of indoor allergens |
|  | - Allergen avoidance is not recommended as a general strategy for people with asthma. - [Evidence A] |
|  | For sensitized patients, although it would seem logical to attempt to avoid allergen exposure in the home, there is some evidence for clinical benefit with single avoidance strategies.  [Evidence A]  and only limited evidence for benefit with multi-component avoidance strategies (in children).  [Evidence B] |
|  | Although allergen avoidance strategies may be beneficial for some sensitized patients[Evidence B]they are often complicated and expensive, and there are no validated methods for identifying those who are likely to benefit.[Evidence D] |
|  | Healthy diet |
|  | - Encourage patients with asthma to consume a diet high in fruit and vegetables for its general health benefits. - [Evidence A] |
|  | Weight reduction for obese patients |
|  | - Include weight reduction in the treatment plan for obese patients with asthma. - [Evidence B] - Increased exercise alone appears to be insufficient. - [Evidence B] |
|  | Breathing exercises |
|  | - - Consider breathing exercises as a supplement to conventional asthma management strategies for symptoms and quality of life, but they do not improve lung function or reduce exacerbation risk.   - [Evidence A] |
|  | Avoidance of food and food chemicals |
|  | - Food avoidance should not be recommended unless an allergy or food chemical sensitivity has been clearly demonstrated - [Evidence D] usually by carefully supervised oral challenges. |
|  | If food allergy is confirmed, food allergen avoidance can reduce asthma exacerbations.  [Evidence D] |
|  | If food chemical sensitivity is confirmed, complete avoidance is not usually necessary, and sensitivity often decreases when overall asthma control improves.[Evidence D] |
|  | Skills training for effective use of inhaler devices |
|  | Check and correct inhaler technique using a standardized checklist.  [Evidence A] |
|  | Asthma information |
|  | Asthma education and training can be delivered effectively by a range of health care providers including pharmacists and nurses.  [Evidence A] |
|  | Asthma education by trained lay health workers has been found to improve patient outcomes and healthcare utilization compared with usual care and to a similar extent as nurse-led education in primary care.[Evidence B] |
|  | Training in guided asthma self-management |
|  | Self-management education dramatically reduces asthma morbidity in adults.  [Evidence A] |
|  | Self-management for asthma reduces unscheduled healthcare use, improves asthma control, is applicable to a wide range of target groups and clinical settings, and does not increase health care costs.[Evidence A] |
|  | Self-monitoring of symptoms and/or peak flow |
|  | For patients carrying out PEF monitoring, use of a laterally compressed PEF chart (showing 2 months landscape format page) allows more accurate identification of worsening asthma than other charts. [Evidence B] |
|  | Written asthma action plans |
|  | The benefits of self-management education for asthma morbidity are greater in adults when action plans include both a step up in ICS and addition of OCS, and for PEF-based plans, when they are based on personal best rather than percent predicted PEF.  [Evidence A] |
|  | The efficacy of is similar regardless of whether patients self-adjust their medications according to an individual written plan or whether medication adjustments are made by a doctor.[Evidence A] |
|  | Managing comorbidities |
|  | Obesity |
|  | ICS are the mainstay of treatment in obese patients.  [Evidence B] |
|  | Weight reduction should be included in treatment plan.  [Evidence B] |
|  | Increased exercise alone appears to be insufficient.  [Evidence B] |
|  | Gastroesophageal reflux disease (GERD) |
|  | In patients with confirmed asthma, consider GERD as a possible cause of a dry cough; however, there is no value in screening patients with uncontrolled asthma for GERD.  [Evidence A] |
|  | Symptomatic reflux should be treated, but patients with poorly controlled asthma should not be treated with anti-reflux therapy unless they also have symptomatic reflux.[Evidence A] |
|  | Exercise-induced bronchoconstriction (EIB) |
|  | Regular controller treatment with ICS significantly reduces EIB.  [Evidence A] |
|  | Training and sufficient warm-up reduce the incidence and severity of EIB.  [Evidence A] |
|  | Taking SABAs, LABAs or chromones prior to exercise prevents EIB  [Evidence A]  but tolerance to protective effects of SABAs and LABAs against EIB develops with regular (more than once-daily) use.  [Evidence A] |
|  | Patients with mild asthma who are prescribed as-needed ICS-formoterol to prevent exacerbations and control symptoms can use same medication prior to exercise, if needed, and do not need to be prescribed a SABA for pre-exercise use.[Evidence B] |
|  | Pregnancy |
|  | Actively treating asthma in pregnancy markedly outweighs any potential risks of usual controller and reliever medications.  [Evidence A] |
|  | ICS reduces risk of exacerbations of asthma during pregnancy  [Evidence A]  and cessation of ICS during pregnancy is a significant risk factor for exacerbations.  [Evidence A] |
|  | Adverse outcomes from exacerbations during pregnancy[Evidence A]including due to lack of ICS or poor adherence, and evidence for safety of usual doses of ICS and LABA [Evidence A]a low priority should be placed on stepping down treatment (however guided) until after delivery [Evidence D]and ICS should not be stopped in preparation for pregnancy or during pregnancy[Evidence C] |
|  | Women – perimenstrual asthma (catamenial asthma) |
|  | Oral contraceptives and/or leukotriene receptor antagonists in addition to the usual strategies for management of asthma may be helpful.  [Evidence D] |
|  | Occupational asthma |
|  | The early identification and elimination of occupational sensitizers and the removal of sensitized patients from any further exposure are important aspects of the management of occupational asthma.  [Evidence A] |
|  | Surgery and asthma |
|  | For elective surgery, meticulous attention should be paid pre-operatively to achieving good asthma control, especially for patients with more severe asthma, uncontrolled symptoms, exacerbation history, or persistent airflow limitation.  [Evidence B] |
|  | Patients taking long-term high dose ICS or who have received OCS for more than 2 weeks during previous 6 months should receive hydrocortisone peri-operatively as they are at risk of adrenal crisis in the context of surgery.[Evidence B] |
|  | Aspirin-exacerbated respiratory disease |
|  | Where an NSAID is indicated for other medical conditions, a COX-2 inhibitor (e.g., celecoxib, or etoricoxib), or paracetamol (acetaminophen), may be considered with appropriate health care provider supervision and observation for at least 2 hours after administration.  [Evidence B] |
|  | ICS are the mainstay of asthma therapy in AERD, but OCS are sometimes required; LTRA may also be useful.[Evidence B] |
|  | Management of worsening asthma and exacerbations |
|  | Combination low dose ICS (budesonide or beclometasone) with formoterol maintenance and reliever regimen.  The combination of rapid-onset LABA (formoterol) and low dose ICS (budesonide or beclometasone) in a single inhaler as both controller and reliever medication is effective in improving asthma symptom control and it reduces exacerbations requiring OCS, and hospitalizations compared with same or higher dose of controller with as- needed SABA reliever.  [Evidence A] |
|  | Other ICS and ICS-LABA maintenance controller regimens |
|  | In self-management, ICS dose at least doubled is associated with improved asthma outcomes and reduced health care utilization.  [Evidence A] |
|  | Temporarily doubling dose of ICS is not effective  [Evidence A]; however, the delay before increasing ICS dose (mean 5–7 days) may contribute. |
|  | In adult patients with an acute deterioration, high dose ICS for 7–14 days (500–1600 mcg BDP-HFA equivalent) has an equivalent benefit to a short course of OCS.  [Evidence A] |
|  | For adults taking combination ICS-LABA as a maintenance controller medication, the ICS dose may be increased by adding a separate ICS inhaler.  [Evidence D] |
|  | Leukotriene receptor antagonists |
|  | For patients with mild asthma using a leukotriene receptor antagonist (LTRA) as their controller, clinician judgment should be used.  [Evidence D] |
|  | Oral corticosteroids |
|  | Written asthma action plan should provide instructions for when and how to commence OCS. Typically, a short course of OCS is used (e.g., 40–50 mg/day usually for 5–7 days (Evidence B) for patients who: Fail to respond to an increase in reliever and controller medication for 2–3 days, deteriorate rapidly or who have a PEF or FEV1 <60% of their personal best or predicted value, and have a history of sudden severe exacerbations.  Patients should contact their doctor if they start taking OCS.  [Evidence D] |
|  | Follow up after a self-managed exacerbation |
|  | Maintenance controller treatment can generally be reduced to previous levels 2–4 weeks after exacerbation  [Evidence D], unless the history suggests that the exacerbation occurred on a background of long-term poorly controlled asthma. |
|  | Inhaled short-acting beta2-agonists |
|  | For mild to moderate exacerbations, repeated administration of inhaled SABA (up to 4–10 puffs every 20 minutes for the first hour) is an effective and efficient way to achieve rapid reversal of airflow limitation.  [Evidence A] |
|  | Delivery of SABA via a pMDI and spacer or a DPI leads to a similar improvement in lung function as delivery via nebulizer.  [Evidence A] |
|  | Systemic corticosteroids |
|  | OCS should be given promptly, especially if patient is deteriorating, or had already increased their reliever and controller medications before presenting  [Evidence B]. Recommended dose of prednisolone for adults is 1 mg/kg/day or equivalent up to a maximum of 50 mg/day. |
|  | OCS should usually be continued for 5–7 days.  [Evidence B] |
|  | Objective assessments |
|  | Oxygen saturation: closely monitor, preferably by pulse oximetry.  [Evidence C] |
|  | Oxygen |
|  | In severe exacerbations, controlled low flow oxygen therapy using pulse oximetry to maintain saturation at 93–95% is associated with better physiological outcomes than with high concentration (100%) oxygen therapy.  [Evidence B] |
|  | Oxygen therapy should not be withheld if pulse oximetry is not available.  [Evidence D] |
|  | Inhaled short-acting beta2-agonists |
|  | Inhaled SABA therapy should be administered frequently for patients presenting with acute asthma. The most cost- effective and efficient delivery is by pMDI with a spacer.  [Evidence A] |
|  | Avoid routine use of intravenous beta2-agonists in patients with severe asthma exacerbations.  [Evidence A] |
|  | Systemic corticosteroids |
|  | Speed resolution of exacerbations and prevent relapse, and in acute care settings should be utilized in all but the mildest exacerbations.  [Evidence A] |
|  | 5- and 7-day courses in adults have been found to be as effective as 10- and 14-day courses respectively [Evidence B] |
|  | There is no benefit in tapering the dose of OCS, either in the short term or over several weeks in patients taking maintenance ICS after discharge.  [Evidence B] |
|  | Inhaled corticosteroids |
|  | Within emergency department: high dose ICS given within first hour after presentation reduces need for hospitalization in patients not receiving systemic corticosteroids.  [Evidence A] |
|  | On discharge home: prescribe ongoing ICS-containing treatment since occurrence of a severe exacerbation is a risk factor for future exacerbations.  [Evidence B] and ICS-containing medications significantly reduce the risk of asthma-related death or hospitalization.  [Evidence A] |
|  | SABA-only treatment of asthma is no longer recommended.  [Evidence B] |
|  | Other treatments |
|  | Ipratropium bromide |
|  | For adults with moderate-severe exacerbations, treatment in emergency department with both SABA and ipratropium, a short-acting anticholinergic, was associated with fewer hospitalizations (Evidence A) and greater improvement in PEF and FEV1 compared with SABA alone.  [Evidence A] |
|  | Magnesium |
|  | Intravenous magnesium sulphate is not recommended for routine use in asthma exacerbations; however, when administered as a single 2 g infusion over 20 minutes, it reduces hospital admissions in some patients, including adults with FEV1 <25–30% predicted at presentation; adults who fail to respond to initial treatment and have persistent hypoxemia.  [Evidence A] |
|  | Addition of intravenous or nebulized magnesium compared with placebo in routine care of asthma exacerbations shows no additional benefit.  [Evidence B] |
|  | Helium oxygen therapy |
|  | There is no role of helium-oxygen compared with air–oxygen in routine care.  [Evidence B] |
|  | Non-invasive ventilation (NIV) |
|  | If NIV is tried, patient should be monitored closely.  [Evidence D] |
|  | It should not be attempted in agitated patients, and patients should not be sedated in order to receive NIV.  [Evidence D] |
|  | Follow up after emergency department presentation or hospitalization for asthma |
|  | Comprehensive intervention programs that include optimal controller management, inhaler technique, and elements of self-management education (self-monitoring, written action plan and regular review) are cost effective and have shown significant improvement in asthma outcomes.  [Evidence B] |
|  | Discharge management after hospital or emergency department care for asthma |
|  | For patients considered at risk of poor adherence, intramuscular corticosteroids may be considered.  [Evidence B] |

**IMPLEMENTAION REMARKS:**

- Ask all patients with adult-onset asthma about their work history and other exposures.
- Encourage patients to use non-polluting heating and cooking sources, and for sources of pollutants to be vented outdoors where possible.
- Encourage patients to identify goals and strategies to deal with emotional stress if it makes their asthma worse.
- Relaxation strategies and breathing exercises may be helpful in reducing asthma symptoms.
- Arrange a mental health assessment for patients with symptoms of anxiety or depression.
- For sensitized patients, closing windows and doors, remaining indoors when pollen and mold counts are highest, and using air conditioning may reduce exposure.
- Athletes should avoid training in extreme cold or pollution.

| AERD | aspirin-exacerbated respiratory disease | LTRA | leukotriene receptor antagonists |
| --- | --- | --- | --- |
| EIB | exercise-induced bronchoconstriction | OCS | oral corticosteroids |
| FeNO | fraction excretion of Nitric Oxide | NIV | non-invasive ventilation |
| FEVI | forced expiratory volume | pMDI: | pressurized metered dose inhaler |
| ICS | inhaled corticosteroids | PEF | Peak expiratory flow |
| LABA | long-acting beta agonist | SLIT | sublingual immunotherapy |
| LAMA | long-acting muscarinic antagonists | SABA | short-acting β-agonist |
| SCIT | subcutaneous immunotherapy | DPI | dry powder inhaler |

**ACRONYMS AND ABBREVIATIONS**

**Guidelines for Diagnosis and Management of COPD
Source Guideline:** Pocket guide to COPD diagnosis, management, and prevention. A guide for health care professionals. 2020 Edition.

**Key to understanding level of evidence and strength of recommendation.**

| Level of Evidence | | | |
| --- | --- | --- | --- |
| Evidence A | Evidence B | Evidence C | Evidence D |
| Randomized controlled trials (RCTs).  Rich body of high-quality evidence without any significant limitation or bias. | Randomized controlled trials (RCTs) with important limitations.  Limited body of evidence. | Non-randomized trials.  Observational studies. | Panel consensus judgement. |

**Table of Recommendations**

|  | Prevention |
| --- | --- |
|  | Influenza vaccination reduces serious illness and death.  [Evidence B]  Influenza covid, pertussis and zoster vaccination is recommended for all patients with COPD.  [Evidence A] |
|  | The 23-valent pneumococcal polysaccharide vaccine (PPSV23) reduces incidence of community-acquired pneumonia in COPD patients aged <65 years with an FEV_1_ <40% predicted and in those with comorbidities.  [Evidence B]  Pneumococcal vaccination: the PCV13 and PPSV23 are recommended for all patients >65 years of age, and in younger patients with significant comorbid conditions including chronic heart or lung disease.  [Evidence B] |
|  | In adults ≥65 years, the 13-valent conjugated pneumococcal vaccine (PCV13) has shown significant efficacy in reducing bacteraemia and serious invasiveness pneumococcal disease.  [Evidence B] |
|  | Treatment |
|  | Inhaled bronchodilators are central to symptom management and commonly given on a regular basis to prevent and reduce symptoms.  [Evidence A] |
|  | Regular and as-needed use of SABA or SAMA improves FEV_1_ and symptoms.  [Evidence A] |
|  | Combinations of SABA and SAMA are superior compared to either medication alone in improving FEV_1_ and symptoms.  [Evidence A] |
|  | LABAs and LAMAs significantly improve lung function, dyspnea, health status, and reduce exacerbation rates.  [Evidence A] |
|  | LAMAs have greater effect on exacerbation of reduction compared with LABAs.  [Evidence A] |
|  | LAMAs have greater effect compared with LABAs on decrease hospitalization.  [Evidence A] |
|  | Combination treatment with a LABA and LAMA increases FEV_1_ and reduces symptoms compared to monotherapy.  [Evidence A] |
|  | Combination treatment with a LABA/LAMA reduces exacerbations compared to monotherapy.  [Evidence B] |
|  | Tiotropium improves effectiveness of pulmonary rehabilitation in increasing exercise performance.  [Evidence B] |
|  | Theophylline exerts a small bronchodilator effect in stable COPD and that is associated with modest symptomatic benefits.  [Evidence A] |
|  | An ICS combined with a LABA is more effective than the individual components in improving lung function, health status and reducing exacerbations in patients with exacerbations and moderate to very severe COPD.  [Evidence A] |
|  | Regular treatment with ICS increases the risk of pneumonia, especially in those with severe disease.  [Evidence A] |
|  | Triple inhaled therapy of LABA/LAMA/ICS improves lung function, symptoms,health status, and reduces exacerbations, compared to LABA/ICS, LABA/LAMA or LAMA monotherapy.  [Evidence A] |
|  | Long-term use of oral glucocorticoids has numerous side effects.  Evidence A] |
|  | Long-term use of oral glucocorticoids has no evidence of benefits.  [Evidence C] |
|  | Chronic Bronchitis |
|  | In patients with severe to very severe COPD and a history of exacerbations:  A PDE4 inhibitor improves lung function and reduces moderate and severe exacerbations. It improves lung function and decreases exacerbations in patients who are on fixed-dose LABA/ICS combinations.  [Evidence A] |
|  | Pharmacological therapies |
|  | Long-term azithromycin and erythromycin therapy reduces exacerbations over one year.  [Evidence A]  Azithromycin is associated with an increased incidence of bacterial resistance.  [Evidence A]  and an increased incidence of hearing test impairment.  [Evidence B] |
|  | Regular treatment with mucolytics such as erdosteine, carbocysteine and NAC reduces risk of exacerbations in select populations.  [Evidence B] |
|  | Observational studies suggest that statins may have positive effects on some outcomes in patients with COPD who receive them for cardiovascular and metabolic indications.  [Evidence C] |
|  | There is no conclusive evidence of a beneficial role of antitussives in patients with COPD.  [Evidence C] |
|  | Vasodilators do not improve outcomes and may worsen oxygenation.  [Evidence B] |
|  | Pulmonary rehabilitation |
|  | Improves dyspnea, health status and exercise tolerance in stable patients.  [Evidence A] |
|  | Reduces hospitalizations in patients who have had a recent exacerbation (≤4 weeks from prior hospitalization).  [Evidence B] |
|  | It leads to a reduction in symptoms of anxiety and depression.  [Evidence A] |
|  | Prevention |
|  | Education alone has not been shown to be effective.  [Evidence C] |
|  | Self-management intervention with communication with a health care professional improves health status and decreases hospitalizations and emergency department visits.  [Evidence B] |
|  | Opiates, neuromuscular electrical stimulation (NMES), oxygen and fans blowing air on the face can relieve breathlessness.  [Evidence C] |
|  | In malnourished patients, nutritional supplementation may improve respiratory muscle strength and overall health status.  [Evidence B] |
|  | Fatigue can be improved by self-management education, pulmonary rehabilitation, nutritional support and mind-body interactions.  [Evidence B] |
|  | Long-term administration of oxygen increases survival in patients with severe chronic resting arterial hypoxemia.  [Evidence A] |
|  | In patients with stable COPD and moderate resting or exercise-induced arterial desaturation, prescription of long-term oxygen does not lengthen time to death or first hospitalization or provide sustained benefit in health status, lung function and 6-minute walk distance.  [Evidence A] |
|  | Resting oxygenation at sea level does not exclude the development of severe hypoxemia when traveling by air.  [Evidence C] |
|  | NPPV may improve hospitalization-free survival in selected patients after recent hospitalization, particularly in those with pronounced daytime persistent hypercapnia (PaCO_2_ ≥ 52 mmHg).  [Evidence B] |
|  | Surgical options |
|  | Lung volume reduction surgery improves survival in severe emphysema patients with an upper-lobe emphysema and low post-rehabilitation exercise capacity.  [Evidence A] |
|  | In selected patients, bullectomy is associated with decreased dyspnea, improved lung function and exercise tolerance.  [Evidence C] |
|  | In appropriately selected patients with very severe COPD, lung transplantation has been shown to improve quality of life and functional capacity.  [Evidence C] |
|  | Advanced emphysema |
|  | In select patients, bronchoscopic interventions reduce end-expiratory lung volume and improve exercise tolerance, health status and lung function at 6-12 months following treatment: Lung coils and vapour ablation.  [Evidence B]  Endobrachial valves  [Evidence B] |
|  | Risk Factor Exposure |
|  | Smoking cessation interventions should be actively pursued in all COPD patients.  [Evidence A] |
|  | Efficient ventilation, non-polluting cooking stoves and similar interventions should be recommended.  [Evidence B] |
|  | Stable COPD |
|  | LABAs and LAMAs are preferred over short-acting agents except for patients with only occasional dyspnea.  [Evidence A] |
|  | Patients may be started on single long-acting bronchodilator therapy or dual long-acting bronchodilator therapy. In patients with persistent dyspnea on one bronchodilator treatment should be escalated to two.  [Evidence A] |
|  | Inhaled bronchodilators are recommended over oral bronchodilators.  [Evidence A] |
|  | Theophylline is not recommended unless other long-term treatment bronchodilators are unavailable or unaffordable.  [Evidence B] |
|  | Long-term monotherapy with ICS is not recommended.  [Evidence A] |
|  | Long-term treatment with ICS may be considered in association with LABAs for patients with a history of exacerbations despite appropriate treatment with long-acting bronchodilators.  [Evidence A] |
|  | Long-term therapy with oral corticosteroids is not recommended.  [Evidence A] |
|  | In patients with severe to very severe airflow limitation, chronic bronchitis and exacerbations the addition of a PDE4 inhibitor to a treatment with long-acting bronchodilators with/without ICS can be considered.  [Evidence B] |
|  | Preferentially, but not only in former smokers with exacerbations despite appropriate therapy, macrolides, in particular azithromycin, can be considered.  [Evidence B] |
|  | Antioxidant mucolytics are recommended only in selected patients.  [Evidence A] |
|  | Drugs approved for primary pulmonary hypertension are recommended for patients with a pulmonary hypertension secondary to COPD.  [Evidence B] |
|  | Consider Low-dose long-acting oral and parenteral opioids for treating dyspnea in COPD patients with severe disease.  [Evidence B] |
|  | Education, Self-management and Pulmonary Rehabilitation |
|  | Education self-management with support of a case manager with or without the use of a written action plan is recommended for prevention of exacerbation complications such as hospital admissions.  [Evidence B] |
|  | Offer rehabilitation to all patients with relevant symptoms and/or a high risk for exacerbation.  [Evidence A] |
|  | Physical activity is a strong predictor of mortality.  [Evidence A] |
|  | Nutritional supplementation should be considered in malnourished patients with COPD.  [Evidence B] |
|  | End of life and Palliative care |
|  | Palliative approaches to symptom control are effective in managing patients with COPD.  [Evidence D] |
|  | End of life care should include discussions with patients and their families about their views on resuscitation, advance directives and place of death preferences.  [Evidence D] |
|  | In patients with severe resting hypoxemia long-term oxygen therapy is indicated.  [Evidence A] |
|  | In patients with stable COPD and resting or exercise-induced moderate desaturation, long-term oxygen treatment should not routinely prescribed. However, individual patient factors may be considered when evaluating the patient’s needs for supplemental oxygen.  [Evidence A] |
|  | Resting oxygenation at sea level does not exclude the development of severe hypoxemia when traveling by air.  [Evidence C] |
|  | In patients with severe chronic hypercapnia and a history of hospitalization for acute respiratory failure, long-term non-invasive ventilation may be considered.  [Evidence B] |
|  | Lung volume reduction surgery should be considered in selected patients with upper-lobe emphysema.  [Evidence A] |
|  | In selected patients with a large bulla surgical bullectomy may be considered.  [Evidence C] |
|  | In select patients with advanced emphysema, bronchoscopic interventions reduce end-expiratory lung volume and improve exercise tolerance, quality of life and lung function at 6-12 months following treatment: Endobronchial valve  [Evidence A] |
|  | In select patients with advanced emphysema, bronchoscopic interventions reduce end-expiratory lung volume and improve exercise tolerance, quality of life and lung function at 6-12 months following treatment: Lung coils  [Evidence A] |
|  | In select patients with advanced emphysema, bronchoscopic interventions reduce end-expiratory lung volume and improve exercise tolerance, quality of life and lung function at 6-12 months following treatment: Vapor ablation  [Evidence B] |
|  | In patients with very severe COPD (progressive disease, BODE score of 7 to 10, and not candidate for lung volume reduction) consider lung transplantation for referral with at least one of the following: (1) history of hospitalization for exacerbation associated with acute hypercapnia (PCO_2_ >50 mmHg); (2) pulmonary hypertension and/or cor pulmonale, despite oxygen therapy; or (3) FEV_1_ <20% and either DLCO <20% or homogenous distribution of emphysema.  [Evidence C] |
|  | Management of Exacerbations |
|  | Systemic corticosteroids can improve lung function (FEV_1_), oxygenation and shorten recovery time and hospitalization duration. Duration of therapy should not be more than 5-7 days.  [Evidence A] |
|  | Antibiotics, when indicated, can shorten recovery time, reduce the risk of early relapse, treatment failure, and hospitalization duration. Duration of therapy should be 5-7 days.  [Evidence B] |
|  | Methylxanthines are not recommended due to increased side effect profiles.  [Evidence B] |
|  | Non-invasive mechanical ventilation should be the first mode of ventilation used in COPD patients with acute respiratory failure who have no absolute contraindication because it improves gas exchange, reduces work of breathing and the need for intubation, decreases hospitalization duration and improves survival.  [Evidence A] |

**Implementation Remarks:**

- Advise patients to avoid continued exposures to potential irritants, if possible.
- Advise that efficient ventilation, non-polluting cooking stoves and similar interventions are beneficial for patients.

**ACRONYMS AND ABBREVIATIONS**

| BODE | Body mass index, airflow Obstruction, Dyspnea, and Exercise Capacity | NMES | neuromuscular electrical stimulation |
| --- | --- | --- | --- |
| COPD | chronic obstructive pulmonary disease | NAC | N-acetyl cysteine |
| DLCO | diffusing capacity of the lungs for carbon monoxide | PCV13 | 13-valent conjugated pneumococcal vaccine |
| FEV | forced expiratory volume | PPSV23 | 23-valent pneumococcal polysaccharide vaccine |
| FVC | forced vital capacity | SABA | short-acting β-agonist |
| ICS | inhaled corticosteroids | SAMA | short-acting muscarinic antagonists |
| LABA | long-acting beta agonist | PDE4 | Phosphodiesterase type 4 |
| LAMA | long-acting muscarinic antagonists |  |  |

**Idiopathic Pulmonary Fibrosis**

**Source Guideline:** 2018 ATS/ERS/JRS/ALAT Clinical Practice Guideline for Diagnosis of Idiopathic Pulmonary Fibrosis Am J Respir Crit Care Med Vol 198, Iss 5, pp e44–e68, Sep 1, 2018, the American Thoracic Society

DOI: 10.1164/rccm.201807-1255ST

**Source Guideline**:2015 ATS/ERS/JRS/ALAT Guidelines for treatment of Idiopathic Pulmonary Fibrosis.Am J Respir Crit Care Med Vol 192, Iss 2, pp e3–e19, Jul 15, 2015, the American Thoracic Society

DOI: 10.1164/rccm.201506-1063ST

**Key to understanding the strength of recommendation and confidence in estimates of effects.**

| **Strength of Recommendation** | | |
| --- | --- | --- |
| **Implication** | **Strong Recommendation** | **Conditional Recommendation** |
| **For Patient** | Most individuals in this situation would want the recommended course of action, and only a small proportion would not. | The majority of individuals in this situation would want the suggested course of action, but many would not. |
| **For Clinicians** | Most individuals should receive intervention. Adherence to this recommendation according to the guideline could be used as a quality criterion or performance indicator. Formal decision aids are not likely to be needed to help individuals make decisions consistent with their values and preferences. | Recognize that different choices will be appropriate for individual patients and that you must help each patient arrive at a management decision consistent with his or her values and preferences. Decision aids may be useful in helping individuals to make decisions consistent with their values and preferences. |
| **For Policy makers** | The recommendation can be adopted as policy in most situations. | Policymaking will require substantial debate and involvement of various stakeholders. |
| **Confidence in estimate of effects** | | |
| Moderate conﬁdence in effect estimates | | |
| Low conﬁdence in effect estimates | | |
| Very low conﬁdence in effect estimates | | |
| Motherhood statement: recommendations for which there is no reasonable alternative to the recommended course of action. | | |

**Table of Recommendations**

|  | Recommendations |
| --- | --- |
|  | Newly diagnosed patients with unknown cause |
|  | For patients who are clinically suspected of having IPF, take a detailed history of both medication use and environmental exposures at home, work, and other places the patient frequently visits to exclude potential causes of the ILD.  [motherhood statement] |
|  | Consider serological testing to aid in the exclusion of CTDs as a potential cause of the ILD.  [motherhood statement] |
|  | Usual Interstitial Pneumonia |
|  | For patients who are clinically suspected of having IPF and have an HRCT pattern of probable UIP, indeterminate for UIP, or an alternative diagnosis, consider performing cellular analysis of their BAL fluid.  [conditional recommendation, very low quality of evidence]   - For patients who are clinically suspected of having IPF and have an HRCT pattern of UIP, consider NOT performing cellular analysis of their BAL fluid.   [conditional recommendation, very low quality of evidence]   - For patients who are clinically suspected of having IPF and have an HRCT pattern of probable UIP, indeterminate for UIP, or an alternative diagnosis, consider SLB.   [conditional recommendation, very low quality of evidence]   - For patients who are clinically suspected of having IPF and have an HRCT pattern of UIP, consider NOT performing SLB.   [strong recommendation, very low quality of evidence]   - For patients who are clinically suspected of having IPF and have an HRCT pattern of UIP, consider NOT performing TBBx.   [strong recommendation, very low quality of evidence]   - For patients who are clinically suspected of having IPF and have an HRCT pattern of UIP, consider NOT performing lung cryobiopsy.   [strong recommendation, very low quality of evidence] |
|  | Newly detected ILD for apparently unknown cause |
|  | Consider multidisciplinary discussion (MDD) for diagnostic decision-making.  [conditional recommendation, very low quality of evidence] |
|  | Avoid measuring serum MMP-7, SPD, CCL-18, or KL-6 for the purpose of distinguishing IPF from other ILDs.  [strong recommendation, very low quality of evidence] |
|  | Recommendations for Specific Treatment |
|  | Do not use warfarin anticoagulation in patients with IPF who do not have a known alternative indication for its use.  [Strong recommendation against, low confidence in estimates of effect] |
|  | Do not use imatinib in patients with IPF.  [Strong recommendation, moderate confidence in estimates of effect] |
|  | Do not use the combination therapy of N-acetylcysteine, azathioprine, and prednisone in patients with IPF.  [Strong recommendation, low confidence in estimates of effect] |
|  | Do not use ambrisentan, a selective ER-A endothelin receptor antagonist, in patients with IPF, regardless of the presence or absence of PH.  [Strong recommendation against, low confidence in estimates of effect] |
|  | Use nintedanib and pirfenidone in patients with IPF.  [Conditional recommendation, moderate confidence in estimates of effect] |
|  | Use regular antiacid treatment for patients with IPF.  [Conditional recommendation, very low confidence in estimates of effect] |
|  | Do not use sildenafil, a phosphodiesterase-5 inhibitor, for the treatment of IPF.  [Conditional recommendation against, moderate confidence in estimates of effect] |
|  | Consider not using bosentan or macitentan, both dual ER-A and ER-B endothelin receptor antagonists, for the treatment of IPF.  [Conditional recommendation against, low confidence in estimates of effect] |
|  | Consider not using N-acetylcysteine monotherapy in patients with IPF.  [Conditional recommendation, low confidence in estimates of effect] |
|  | The committee did not make a recommendation regarding single versus bilateral lung transplantation in patients with IPF.  [The committee acknowledged that further evidence is needed and should be evaluated to guide this clinical decision] |
|  | The committee did not make a recommendation regarding treatment of PH in patients with IPF.  [The committee acknowledged that further evidence is needed and should be evaluated to guide this clinical decision] |

**ACRONYMS & ABBREVIATIONS**

| BAL | Bronchoalveolar lavage | HRCT | High-Resolution Computed Tomography |
| --- | --- | --- | --- |
| CTDs | Connective Tissue Diseases | KL-6 | Krebs von den Lungen-6 |
| CT | Computed Tomography | MDD | Multidisciplinary Discussion |
| COPD | Chronic Obstructive Pulmonary Disease | MMP-7 | Matrix Metalloproteinase-7 |
| CCL-18 | Chemokine (C-C motif) ligand 18 | TBBX | Transbronchial Lung Biopsy |
| ER-A | Endothelin Receptor Antagonist, | UIP | Usual Interstitial Pneumonia |
| ILD | Interstitial Lung Disease | IPF | Idiopathic Pulmonary Fibrosis |
| SLB | Surgical Lung Biopsy | PH | Pulmonary Hypertension |
| SPD | Surfactant Protein D |  |  |

**Management of Bronchiectasis**

**Source Guideline:** British Thoracic Society Guidelines for Bronchiectasis in Adults, Thorax; An international journal of Respiratory medicine volume 74, supplement 1, January 2019

**Key to understanding level of evidence and strength of recommendation.**

| Level of Evidence | |
| --- | --- |
| Grade | Evidence |
| 1++ | High quality meta-analyses, systematic reviews of RCTs, or RCTs with a very low risk of bias |
| 1+ | Well conducted meta-analyses, systematic reviews of RCTs, or RCTS with a low risk of bias |
| 1- | Meta-analyses, systematic reviews of RCTs, or RCTs with a high risk of bias |
| 2++ | High quality systematic reviews of case-control or cohort studies or high-quality case-control or cohort studies with a very low risk of confounding, bias or chance and a high probability that the relationship is casual |
| 2+ | Well conducted case-control or cohort studies with a low risk of confounding, bias or chance and a moderate probability that the relationship is casual |
| 2- | Case-control or cohort studies with a high risk of confounding, bias or chance and a significant risk that the relationship is not casual |
| 3 | Non-analytic studies, for example, case reports, case series |
| 4 | Expert opinion |
| Grades of Recommendation | |
| Grade | Type of Evidence |
| A | At least one meta-analysis, systematic review, or RCT rated as 1++and directly applicable to the target population or A systematic review of RCTs or a body of evidence consisting principally of studies rated as 1+directly applicable to the target population and demonstrating overall consistency of results |
| B | A body of evidence including studies rated as 2++directly applicable to the target population and demonstrating overall consistency of results or Extrapolated evidence from studies rated as 1++or 1+ |
| C | A body of evidence including studies rated as 2+directly applicable to the target population and demonstrating overall consistency of results or Extrapolated evidence from studies rated as 2++ |
| D | Evidence of level 3 or four or Extrapolated evidence from studies rates as 2+ |

**Table of Recommendations**

|  | Diagnosis of bronchiectasis |
| --- | --- |
|  | Consider investigation for bronchiectasis in patients with:   - Persistent production of mucopurulent or purulent sputum particularly with relevant associated risk factors.   [D]   - Rheumatoid arthritis if they have symptoms of chronic productive cough or recurrent chest infections.   [C]   - Chronic Obstructive Pulmonary Disease (COPD) with frequent exacerbations (two or more annually) and a previous positive sputum culture for *P. aeruginosa* whilst stable.   [B]   - Inflammatory bowel disease and chronic productive cough.   [C] |
|  | Determining the diagnosis of bronchiectasis |
|  | Perform baseline chest X-ray in patients with suspected bronchiectasis.  [D]  Perform baseline imaging during clinically stable disease as this is optimal for diagnostic  and serial comparison purposes.  [D] |
|  | Perform a high-resolution computed tomography (HRCT) scan of the chest to confirm a diagnosis of bronchiectasis when clinically suspected.  [C] |
|  | Investigations |
|  | A panel of investigations should be performed to establish the underlying cause of bronchiectasis.  [B] |
|  | Co-morbidities and past medical history should be recorded in patients diagnosed with bronchiectasis to identify relevant and possibly causative disease such as rheumatoid arthritis, COPD, asthma, gastro-esophageal reflux disease and inflammatory bowel disease.  [C] |
|  | Measure full blood count, serum total IgE and assessment of sensitisation (specific IgE or skin prick test) to *Aspergillus fumigatus* in all patients with bronchiectasis.  [D] |
|  | Serum Immunoglobulin G (IgG), Immunoglobulin A (IgA) and Immunoglobulin M (IgM) should be performed in all patients with bronchiectasis.  [C] |
|  | Test for cystic fibrosis in patients with supporting clinical features, for example, early onset, male infertility, malabsorption, pancreatitis.  [B] |
|  | Sputum cultures should be performed in all patients with bronchiectasis for routine and mycobacterial culture.  [D] |
|  | Consider measuring baseline specific antibody levels against capsular polysaccharides of *Streptococcus pneumoniae* in all patients to investigate for specific antibody deficiency. If pneumococcal antibodies are low, immunize with 23 valent polysaccharide pneumococcal vaccine, followed by measurement of specific antibody levels 4–8 weeks later.  [D] |
|  | Test for Primary Ciliary Dyskinesia (PCD) in patients with supporting clinical features, including a history of neonatal distress, symptoms from childhood, recurrent otitis media, rhinosinusitis, or infertility. [A] |
|  | STABLE STATE TREATMENT  Airway clearance techniques |
|  | Teach individuals with bronchiectasis to perform airway clearance.  [D] |
|  | Offer active cycle of breathing techniques or oscillating positive expiratory pressure to individuals with bronchiectasis.  [D] |
|  | Consider gravity assisted positioning (where not contraindicated) to enhance the effectiveness of an airway clearance technique.  [D] |
|  | Mucoactives in bronchiectasis |
|  | Do NOT use recombinant human DNase in adults with Non CF-bronchiectasis.  [A] |
|  | Consider the use of humidification with sterile water or normal saline to facilitate airway clearance.  [D] |
|  | Long term anti-inflammatory therapies in bronchiectasis |
|  | Do NOT routinely offer inhaled corticosteroids to patients with bronchiectasis without other indications (such as ABPA, chronic asthma, COPD and inflammatory bowel disease).  [B] |
|  | Do NOT offer long-term oral corticosteroids for patients with bronchiectasis without other indications (such as ABPA, chronic asthma, COPD, inflammatory bowel disease).  [D] |
|  | Do NOT routinely offer phosphodiesterase type 4 (PDE4) inhibitors, methylxanthines or leukotriene receptor antagonists for bronchiectasis treatment.  [D] |
|  | Do NOT routinely offer CXCR2 antagonists, neutrophil elastase inhibitors or statins for bronchiectasis treatment.  [B] |
|  | Treatment |
|  | Consider long term antibiotics in patients with bronchiectasis who experience 3 or more exacerbations per year.  [A] |
|  | For P. aeruginosa colonised patients |
|  | Use inhaled colistin OR inhaled gentamicin for patients with bronchiectasis and chronic *Pseudomonas aeruginosa* infection.  [B] |
|  | Consider azithromycin as an alternative (eg, if a patient does not tolerate inhaled antibiotics) to an inhaled antibiotic for patients with bronchiectasis and chronic *P. aeruginosa* infection.  [B] |
|  | Consider azithromycin as an additive treatment to an inhaled antibiotic for patients with bronchiectasis and chronic *P. aeruginosa* infection who have a high exacerbation frequency.  [D] |
|  | For non-P. aeruginosa colonised patients |
|  | Use azithromycin for patient with bronchiectasis.  [A] |
|  | Consider inhaled gentamicin as a second line alternative to azithromycin.  [B] |
|  | Consider doxycycline as an alternative in patients intolerant of macrolides or in whom they are ineffective.  [C] |
|  | Long-term bronchodilator treatment |
|  | Use of bronchodilators in patients with bronchiectasis and co-existing COPD or asthma should follow the guideline recommendations for COPD or asthma.  [D] |
|  | Offer a trial of long-acting bronchodilator therapy in patients with symptoms of significant breathlessness. [D] |
|  | Reversibility testing to beta 2 agonist or anticholinergic bronchodilators may help to identify patients with co-existing asthma but there is no evidence to suggest that a response is required in order to benefit from bronchodilators.  [D] |
|  | Pulmonary rehabilitation |
|  | Offer pulmonary rehabilitation to individuals who are functionally limited by shortness of breath (Modified Medical Research Council (MMRC) Dyspnea Scale ≥ 1). Consider the use of inspiratory muscle training in conjunction with conventional pulmonary rehabilitation to enhance the maintenance of the training effect.  [B] |
|  | Role of surgery |
| Refer  to  Specialist | Consider lung resection in patients with localized disease whose symptoms are not controlled by medical treatment optimized by a bronchiectasis specialist.  [D] |
| Refer  to  Specialist | Offer multidisciplinary assessment, including a bronchiectasis physician, a thoracic surgeon, and an experienced anesthetist, of suitability for surgery and pre-operative assessment of cardiopulmonary reserve post resection.  [D] |
|  | Lung transplantation for bronchiectasis |
| Refer  to  Specialist | Consider transplant referral in bronchiectasis patients aged 65 years or less if the FEV1 is <30% with significant clinical instability or if there is a rapid progressive respiratory deterioration despite optimal medical management.  [D] |
| Refer  to  Specialist | Consider earlier transplant referral in bronchiectasis patients with poor lung function and the following additional factors: massive hemoptysis, severe secondary pulmonary hypertension, ICU admissions or respiratory failure (particularly if requiring NIV).  [D] |
|  | Role of influenza and pneumococcal vaccination |
|  | Offer pneumococcal vaccination and annual influenza immunization to all patients with bronchiectasis. [D] |
|  | Treatment of respiratory failure |
|  | Consider long term oxygen therapy for patients with bronchiectasis and respiratory failure, using the same eligibility criteria as for COPD.  [D] |
|  | Consider domiciliary non-invasive ventilation with humidification for patients with bronchiectasis and respiratory failure associated with hypercapnia, especially where this is associated with symptoms or recurrent hospitalisation.  [D] |
|  | Bronchiectasis and other treatments |
|  | Do not routinely recommend alternative treatments (for example cough suppression, nutritional supplementation, complementary therapy/homeopathy, supplemental treatments) as part of the management of patients with bronchiectasis.  [D] |
|  | Impact of pathogens on prognosis in bronchiectasis |
|  | Consider patients with chronic *P. aeruginosa* colonization at higher risk of bronchiectasis-related complications.  [B] |
|  | Perform regular sputum microbiology screening for patients with clinically significant bronchiectasis to monitor for pathogens and detect new isolation of *P. aeruginosa.*  [C] |
|  | Eradication of potentially pathogenic microorganisms |
|  | Offer patients with bronchiectasis associated with clinical deterioration and a new growth of *P. aeruginosa* (1st isolation or regrowth in the context of intermittently positive cultures) eradication antibiotic treatment. (first line treatment: ciprofloxacin 500–750 mg bd for 2 weeks; second line treatment: iv antipseudomonal beta-lactam ± an iv aminoglycoside for 2 weeks, followed by a 3-month course of nebulized colistin, gentamicin or tobramycin).  [D] |
|  | Discuss with patients the potential risks and benefits of starting eradication antibiotic treatment versus clinical observation following a new growth of *P. aeruginosa* in the context of stable bronchiectasis. This will include consideration of the likelihood of achieving sustained eradication, the risk of developing chronic infection, and the risk of adverse events with each management approach.  [D] |
|  | Offer patients with bronchiectasis associated with clinical deterioration and a new growth of methicillin-resistant *S. aureus* (MRSA) (1st isolation or regrowth in the context of intermittently positive cultures) eradication. This should be attempted especially in view of infection control issues.  [D] |
|  | Treatments to improve outcomes in patients with bronchiectasis and allergic broncho-pulmonary aspergillosis |
|  | Offer oral corticosteroid to patients with active ABPA. An initial dose of 0.5 mg/kg/d, for 2 weeks is recommended. Wean steroids according to clinical response and serum IgE levels.  [D] |
|  | Consider itraconazole as a steroid sparing agent for patients dependent on oral corticosteroids where difficulty in weaning is experienced.  [B] |
|  | Monitor patients with active ABPA with total IgE level to assess treatment response.  [C] |
|  | Does immunoglobulin replacement treatment therapy improve outcomes in patients with bronchiectasis due to antibody deficiency |
|  | Offer IgG therapy to all patients with common variable immune deficiency (CVID) and x linked agammaglobulinemia (XLA).  [B] |
|  | Consider IgG therapy for patients with specific polysaccharide antibody deficiency and/or IgA deficiency or IgG subclass deficiencies with absent/impaired pneumococcal vaccine antibody responses who continue to have objective evidence of bacterial sino-pulmonary infection and progressive disease despite appropriate management of bronchiectasis.  [C] |
|  | Gastro-esophageal reflux disease (GERD) and bronchiectasis |
|  | Treat GERD symptoms in patients with bronchiectasis.  [D] |
|  | Prevalence of rhinosinusitis in patients with stable bronchiectasis |
|  | The evaluation of patients with bronchiectasis should include assessment of symptoms of chronic rhinosinusitis. Patients with bronchiectasis and symptoms of rhinosinusitis should be evaluated and treated according to existing evidence-based treatment pathways.  [D] |
|  | Treatment of bronchiectasis in the presence of co-morbidities |
|  | Consider a trial of inhaled and/or oral corticosteroids in patients with bronchiectasis and inflammatory bowel disease (IBD).  [D] |
|  | Ensure optimal control of asthma and allergies in patients with both bronchiectasis and asthma  [D]. |
|  | Monitor patients with co-morbid COPD and bronchiectasis as they are at higher risk of death.  [D] |
|  | Patients with bronchiectasis and autoimmune conditions should be carefully assessed for autoimmune related lung disease and often require long term follow up in a secondary care setting.  [D] |
|  | Patients with bronchiectasis who require disease modifying antirheumatic drugs (DMARDs) or biologics for rheumatoid arthritis should be referred to a chest physician for further assessment before treatment is started.  [D] |
|  | Monitoring bronchiectasis |
|  | All patients with bronchiectasis should undergo routine monitoring in order to identify disease progression, pathogen emergence and modify treatment where necessary.  [D] |
|  | Cross-infection with pathogenic organisms |
|  | Individual or cohort segregation based on respiratory tract microbiology results are not routinely required for patients with bronchiectasis.  [D] |

**ACRONYMS AND ABBREVIATIONS**

| ABPA | Allergic Bronchopulmonary Aspergillosis | HRCT | High Resolution Computerized Tomography |
| --- | --- | --- | --- |
| COPD | Chronic Obstructive Pulmonary Disease | MMRC | Modified Medical Research Council |
| CF | Cystic Fibrosis | NIV | Non-invasive ventilation |
| CVID | Common Variable Immune Deficiency | CXCR2 | Oral chemokine receptor 2 |
| CTD | Connective Tissue Disease | PCD | Primary Ciliary Dyskinesia |
| DMARDs | Disease Modifying Anti-Rheumatic Drugs | PDE4 | Phosphodiesterase type 4 |
| FEVI | Forced expiratory volume-1 | XLA | X-linked Agammaglobulinemia (XLA |
| GERD | Gastroesophageal reflux disease |  |  |

**References**

1. GRADEpro G. GRADEpro GDT: GRADEpro Guideline Development Tool [Software]. McMaster University, 2015 (developed by Evidence Prime, Inc.). 2015.

2. Griffiths P. Evidence informing practice: introducing the mini-review. British journal of community nursing. 2002;7:38-9.
