# Supplementary material for: GRADE-ADOLOPMENT of clinical practice guidelines and creation of clinical pathways for the primary care management of chronic respiratory conditions in Pakistan
Source: BMC Pulm Med. 2023 Apr 17;23:123. doi: 10.1186/s12890-023-02409-4 (PMC10111762; doi:10.1186/s12890-023-02409-4)

Supplementary Figure 1: Primary Care Clinical Diagnosis and Referral Pathway for Idiopathic Pulmonary Fibrosis (IPF)

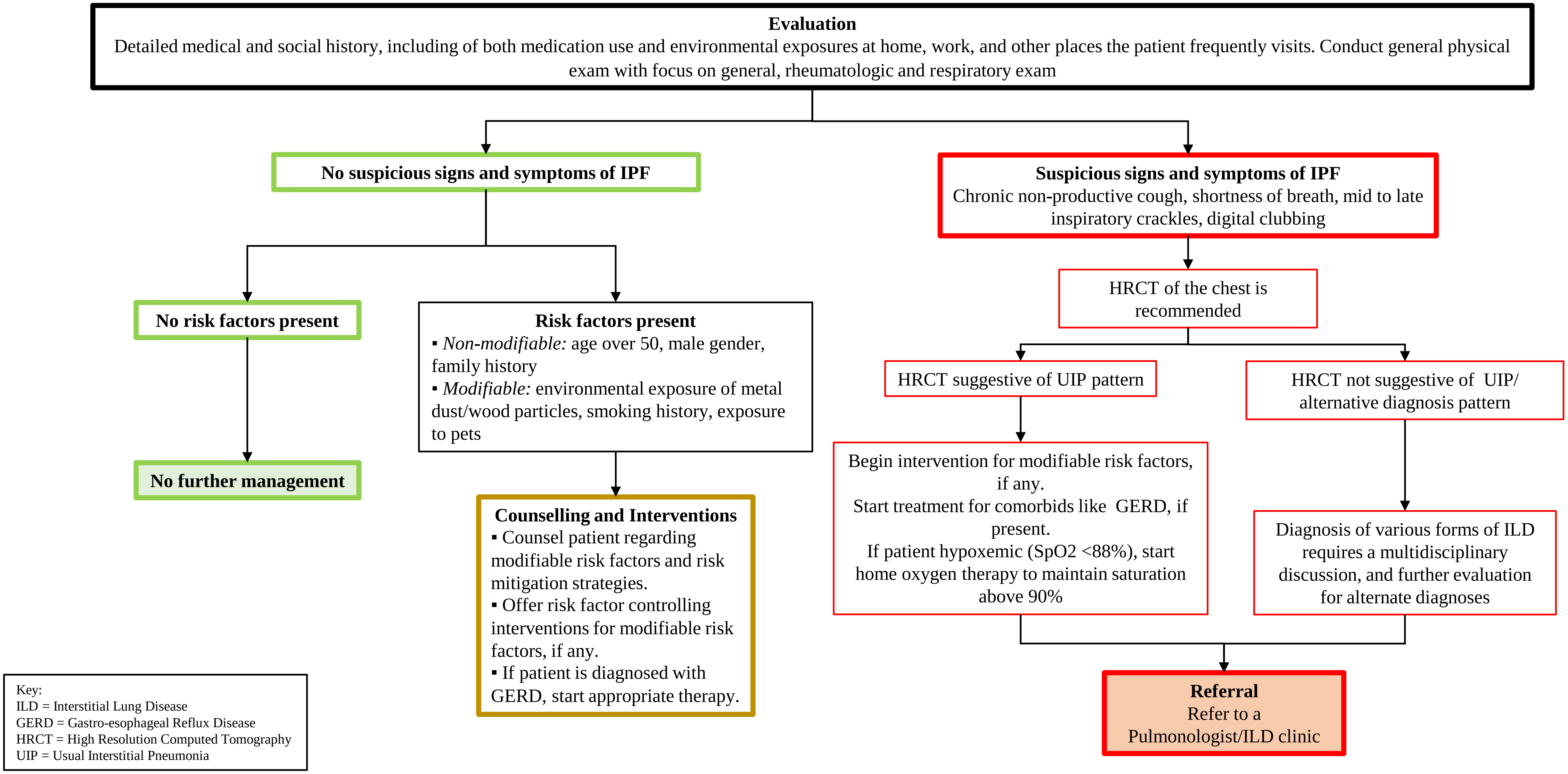

Supplementary Figure 2: Primary Care Clinical Diagnosis and Referral Pathway for Bronchiectasis

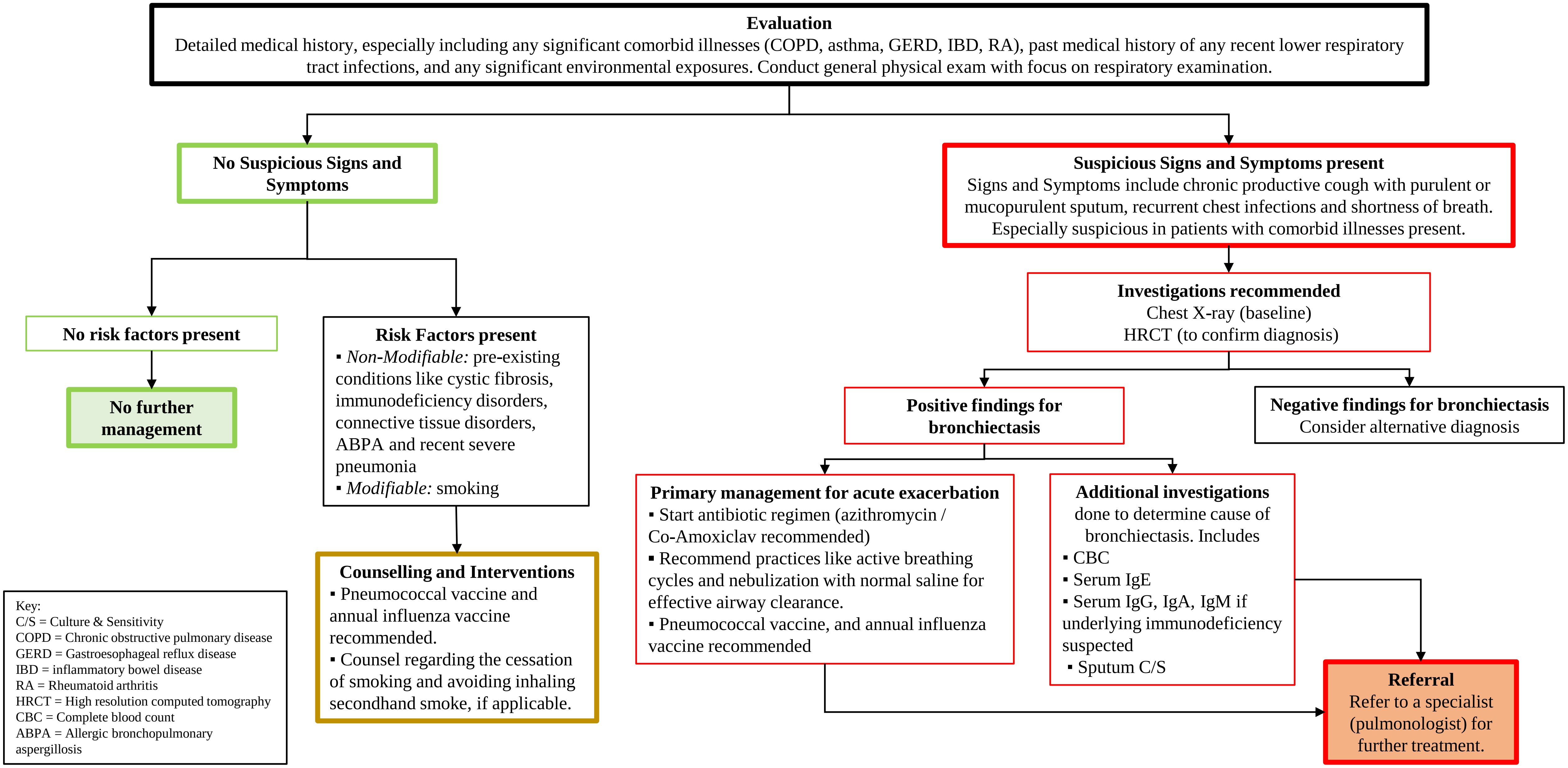

Supplementary Figure 3: Primary Care Clinical Diagnosis and Referral Pathway for Chronic Obstructive Pulmonary Disease (COPD)

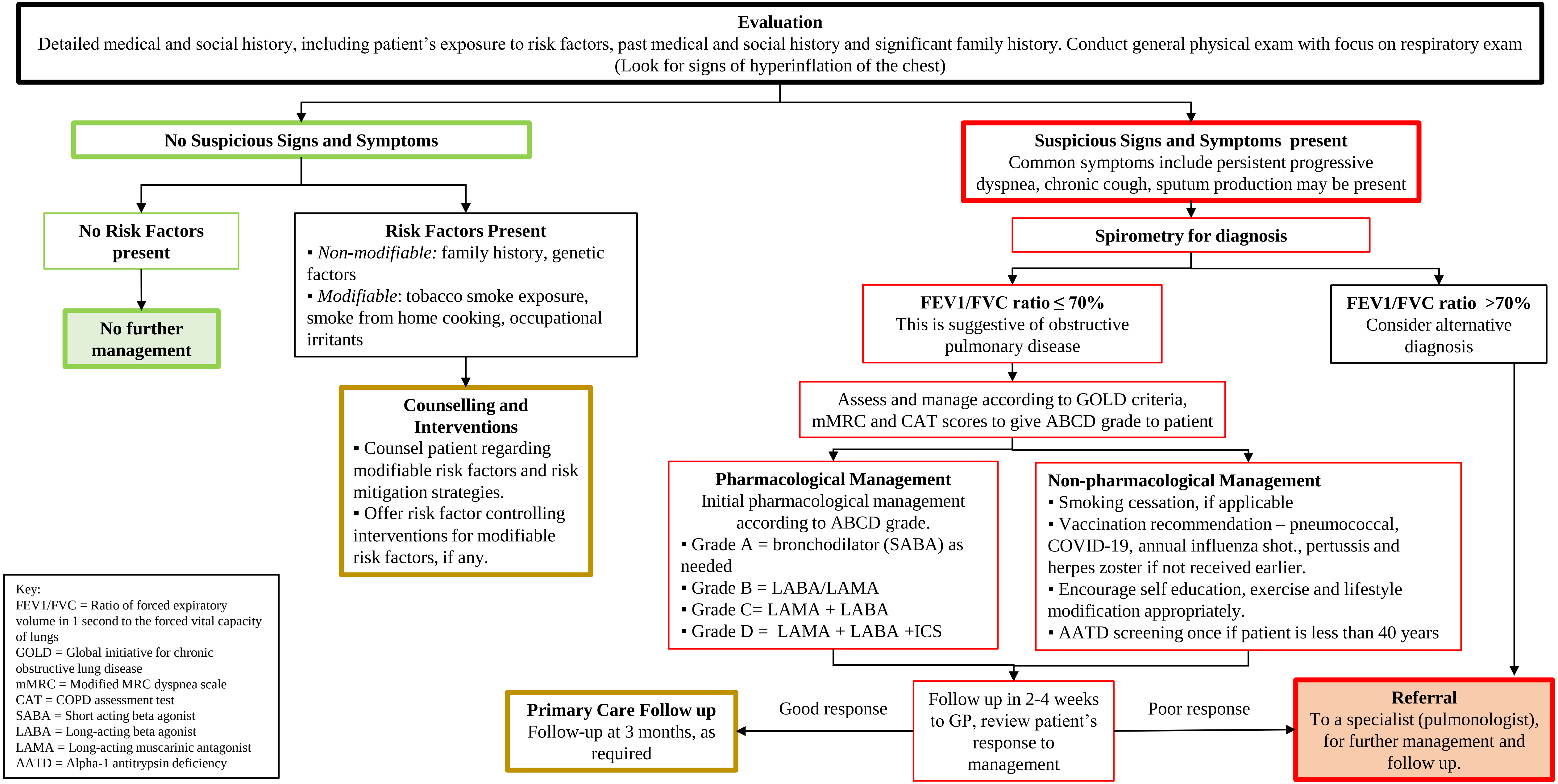

Supplementary Figure 4: Primary Care Clinical Diagnosis and Referral Pathway for Asthma

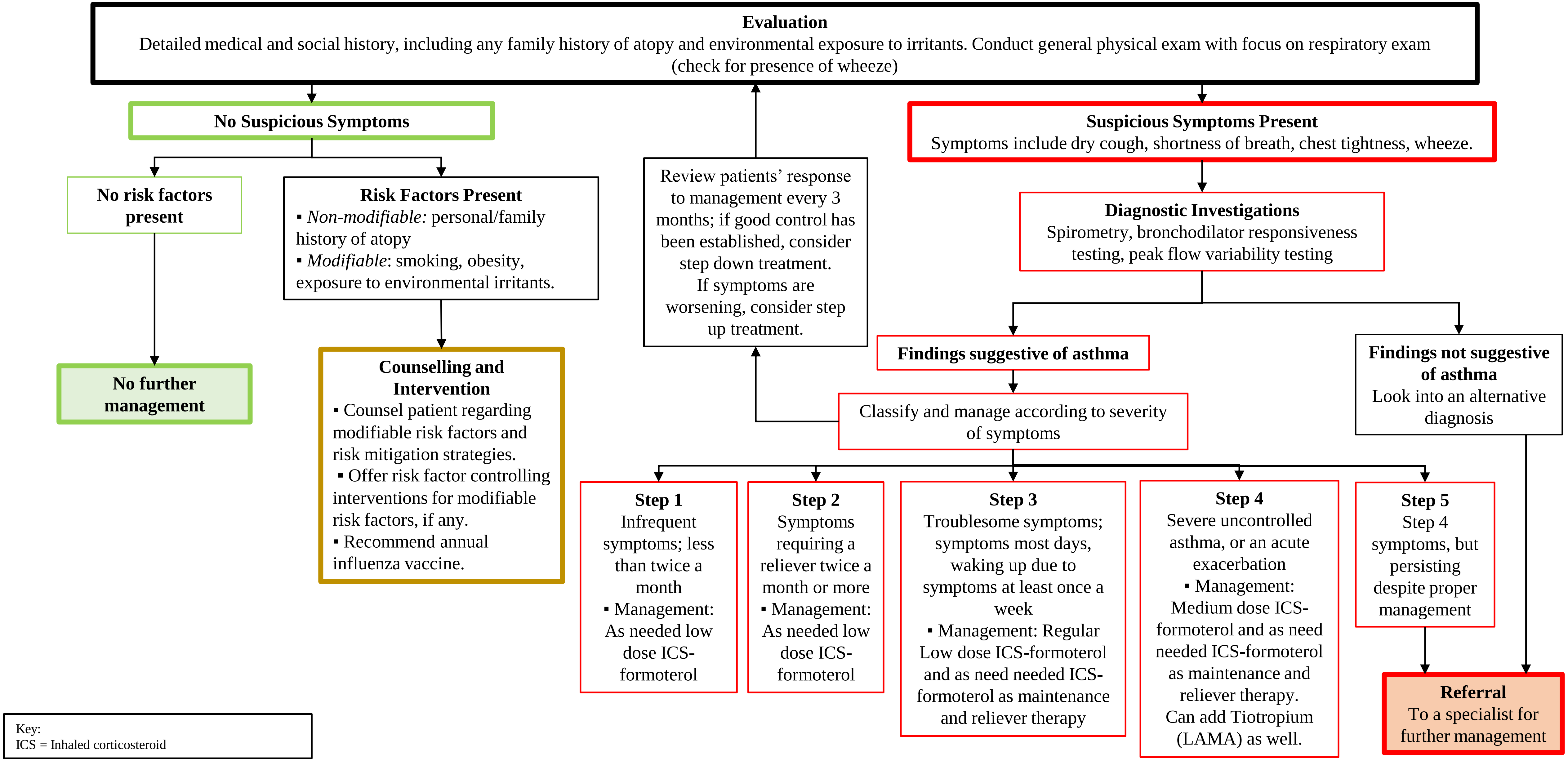

Supplement: Supplementary file 2 — Supplementary Material 2 [file 12890_2023_2409_MOESM2_ESM.pdf]
